# Development and validation of a hypertension risk prediction model and construction of a risk score in a Canadian population

**DOI:** 10.1038/s41598-022-16904-x

**Published:** 2022-07-27

**Authors:** Mohammad Ziaul Islam Chowdhury, Alexander A. Leung, Khokan C. Sikdar, Maeve O’Beirne, Hude Quan, Tanvir C. Turin

**Affiliations:** 1 Department of Community Health Sciences, University of Calgary, 3280 Hospital Drive NW, Calgary, AB T2N 4Z6 Canada; 2 Department of Family Medicine, G012F, Health Sciences Centre, Cumming School of Medicine, University of Calgary, 3330 Hospital Drive NW, Calgary, AB T2N 4N1 Canada; 3 Department of Psychiatry, University of Calgary, 3280 Hospital Drive NW, Calgary, AB T2N 4Z6 Canada; 4 Department of Medicine, University of Calgary, 3280 Hospital Drive NW, Calgary, AB T2N 4Z6 Canada; 5 Health Status Assessment, Surveillance and Reporting, Public Health Surveillance and Infrastructure, Provincial Population and Public Health, Alberta Health Services, 10101 Southport Rd. SW, Calgary, AB T2W 3N2 Canada

**Keywords:** Diseases, Medical research, Risk factors

## Abstract

Identifying high-risk individuals for targeted intervention may prevent or delay hypertension onset. We developed a hypertension risk prediction model and subsequent risk sore among the Canadian population using measures readily available in a primary care setting. A Canadian cohort of 18,322 participants aged 35–69 years without hypertension at baseline was followed for hypertension incidence, and 625 new hypertension cases were reported. At a 2:1 ratio, the sample was randomly divided into derivation and validation sets. In the derivation sample, a Cox proportional hazard model was used to develop the model, and the model's performance was evaluated in the validation sample. Finally, a risk score table was created incorporating regression coefficients from the model. The multivariable Cox model identified age, body mass index, systolic blood pressure, diabetes, total physical activity time, and cardiovascular disease as significant risk factors (*p* < 0.05) of hypertension incidence. The variable sex was forced to enter the final model. Some interaction terms were identified as significant but were excluded due to their lack of incremental predictive capacity. Our model showed good discrimination (Harrel’s C-statistic 0.77) and calibration (Grønnesby and Borgan test, $\chi^{2}$ statistic = 8.75, *p* = 0.07; calibration slope 1.006). A point-based score for the risks of developing hypertension was presented after 2-, 3-, 5-, and 6 years of observation. This simple, practical prediction score can reliably identify Canadian adults at high risk of developing incident hypertension in the primary care setting and facilitate discussions on modifying this risk most effectively.

## Introduction

Hypertension, which affects more than 1 in 5 Canadians^1^, is a common medical condition and is the leading modifiable risk factor for preventable cardiovascular morbidity and mortality^2^. Hypertension prevention and blood pressure management in hypertensive patients is considered a major public health concern^3^. For decades, the focus of interventions has been on improving hypertension detection, treatment, and control, but relatively little work has been done to promote primary prevention. Evidence suggests that the risk of progression to hypertension depends on several factors. Older age, female sex, increased body mass index (BMI), family history of hypertension, premature cardiovascular disease, sedentary lifestyles, unhealthy diet, and high sodium consumption are among the factors reported as significant predictors of hypertension^4^.

Screening people at greater risk of hypertension opens the possibility of promoting individualized preventive initiatives because we will know who to target, what to target, where to target, and how to target^5,6^. A prediction model helps screen high-risk individuals by estimating their probability of developing hypertension within a particular time^7^. Over the past decades, many prediction models have been developed in different populations to predict incident hypertension^8–15^, but their performance in accurately forecasting it varies. To the best of our knowledge, prediction models for the risk of incident hypertension that directly address the Canadian population have not yet been established. One method for predicting the risk of developing hypertension in Canadian populations was to use an existing model and evaluate its performance through external validation of the model. However, in light of the following considerations, we opted to construct a new model rather than externally validate an existing model. First, prediction models are determined by an equation that includes risk factors, risk coefficients (multiplying factors that assign an etiological weight to single factors), and the general population's survival probability or baseline risk without the disease^16^. These elements vary depending on the type of population, especially when very different cultures are compared (i.e., European countries and Asian countries). Second, each population has a different risk of contracting the disease, and each population may have a different distribution of risk factors that weigh differently in determining the disease^16^. Furthermore, the disease may occur with varying probability, resulting in a different survival rate without it. Third, heterogeneity in predictor effects (the same predictor may have different prognostic values in different populations), differences in outcome incidence, and differences in case-mix between the development and validation cohorts can all have a significant impact on a model's predictive performance and frequently result in poor performance when applied to a different population^17–19^. Furthermore, many existing models were restricted to people of a specific ethnicity or those who were already at high risk, or only included a limited number of clinical variables^4^. Because of these facts, the performance of a prediction model can vary significantly by population. As a result, the prediction model's accuracy is frequently acceptable for that index population but is not necessarily generalizable to populations other than the one for which the model was developed^16^. We assessed this by applying a few published hypertension prediction models to our population and comparing their predictive performance. Prediction models cannot be transferred directly from one population type to another^20–22^. The lack of a hypertension risk index specific to the Canadian population prompted us to create a new hypertension prediction model using one of Canada's largest cohort studies, which will aid local clinicians and healthcare providers in clinical decision-making, planning, and proper management of hypertension-related healthcare services.

Faced with the lowest national rates of blood pressure control in over a decade, effective strategies to identify Canadians at the highest risk of developing high blood pressure to prevent the onset of hypertension have become more relevant than ever^1^. To this end, we created and internally validated a simple and practical risk prediction model for incident hypertension in the Canadian adult population. We also derived the point-based risk score from the developed model to facilitate clinical practice use for decision-making.

## Methods

### Study population

The study subjects were from Alberta’s Tomorrow Project (ATP) cohort data. ATP is a province-wide prospective cohort study and consists of Alberta’s residents, aged 35–69 years, without any history of cancer, other than non-melanoma skin cancer^23^. ATP contains baseline and longitudinal information on socio-demographic characteristics, personal and family history of the disease, medication use, lifestyle and health behavior, environmental exposures, and physical measures. ATP was designed to be representative of healthy middle-aged adults in Alberta. A more detailed description of ATP and its recruitment process is provided in the supplementary material (Appendix 1).

Our study cohort consists of 25,359 participants who completed ATP’s CORE questionnaire and consented to have their data linked with Alberta’s administrative health data. Linking with administrative health data was done to establish the necessary longitudinal follow-up to determine hypertension incidence. We excluded 6996 participants from the analysis who had hypertension at baseline and did not meet eligibility criteria (i.e., free of hypertension at baseline). We also excluded 41 participants who responded to hypertension status questions at baseline as “don’t know” or “missing”. Eighteen thousand three hundred twenty-two participants were included in the final analysis.

### Selection of candidate variables

Before commencing the analysis, we compiled a list of available potential candidate variables. We determine the possible candidate variables for inclusion in model development based on a literature search^4,24^, variables that have been used in the past^25^, and discussion with content experts. For this study, we considered 29 candidate variables for inclusion in the model. We deliberately did not consider genetic risk factors/biomarkers as potential candidate variables given our model’s intended clinical application. Inclusion of the genetic risk factors in the model can reduce the model’s usability due to a lack of readily available information.

### Definition of outcome and variables

The outcome incident hypertension was determined from linked administrative health data using a coding algorithm. We used the relevant ICD-9 and ICD-10 codes (ICD-9-CM codes: 401.x, 402.x, 403.x, 404.x, and 405.x; ICD-10-CA/CCI codes: I10.x, I11.x, I12.x, I13.x, and I15.x) and a validated hypertension case definition (two physician claims within two years or one hospital discharge for hypertension) to define hypertension incidence (sensitivity 75%, positive predictive value 81%)^26^.

Out of 29 candidate variables, 11 were continuous, and 18 were categorical. Continuous variables remained continuous in the model developed and categorized only for deriving risk scores. A detailed description of the variables and their categorization is provided in the supplementary material (Appendix 2).

### Missing values

Our dataset has missing values on several candidate variables ranging from 0 to 26%. Information on missing values for different candidate variables is presented in the supplementary table (Table S1). We used multiple imputation for missing data^27^. This technique predicts the missing values by utilizing the existing information from other available variables^28^ and then substitute the missing values with the predicted values to create a complete dataset. Multiple imputation by chained equations (MICE) was used to impute the missing values using Stata’s “ice” command^29^.

### Statistical analysis

Before imputing missing values, the required assumption “missing at random” for performing multiple imputations was checked. We compared the study characteristics of those with missing with those without missing information using appropriate tests (unpaired t-test or the χ^2^-test). Continuous variables were expressed as the mean (SE), and categorical variables were expressed as numbers (percentage of the total). We randomly split subjects into two sets: the derivation set, which included 67% (two-thirds) of the sample (n = 12,233), and the validation set, which included the remaining 33% (one-third) (n = 6089). The two groups’ baseline characteristics were compared using the unpaired t-test or the χ^2^-test, as appropriate. We developed a risk prediction model from the derivation data using the multivariable Cox proportional hazards model and assessed the goodness of fit using the validation data.

Collinearity among the variables was tested using the variance inflation factor (VIF) with a threshold of 2.5^30^. From the list of candidate variables, highly correlated variables were excluded based on VIF before applying the model.

The univariate Cox proportional hazards model was applied first to screen the variables for a significant association (*p* < 0.20)^31^ with hypertension incidence in the derivation set. Variables identified as significant in the univariate association were later put into a multivariable Cox proportional hazards model to determine ultimate significant risk factors (*p* < 0.05) of incident hypertension. The interaction terms were also tested, with significant variables identified in the multivariable Cox model. During the model development process, the proportional hazard assumption associated with the Cox model was also tested. There are several methods for verifying proportionality assumption, and we tested the proportionality assumption by using the Schoenfeld and scaled Schoenfeld residuals. We tested the proportionality of the model as a whole and proportionality for each predictor.

The following general equation was used to calculate the risk of incident hypertension within time $t$:

$$ Probability = 1 - S_{0} \left( t \right)^{{{\text{exp}}\left( {\mathop \sum \limits_{i = 1}^{p} \beta_{i} X_{i} - \mathop \sum \limits_{i = 1}^{p} \beta_{i} \overline{X}_{i} } \right)}} $$

where $S_{0} \left( t \right)$ is the baseline survival function, assuming all variables are represented by average values at follow-up time $t$; $\beta_{i}$ is the estimated regression coefficient of the $i$th variable; $X_{i}$ is the value of the $i$th variable; $\overline{X}_{i}$ is the corresponding mean, and $p$ denotes the number of variables.

In the validation data, the model’s predictive performance was assessed. Model discrimination was evaluated using Harrell’s C-statistic^32^. Harrel’s C-statistic indicates the proportion of all pairs of subjects that can be ordered such that the subject who survived longer will have the higher predicted survival time than the subjects who survived shorter, assuming that these subject pairs are selected at random. Calibration was assessed using the Grønnesby and Borgan (GB) test^33^. The GB test is an overall goodness-of-fit test for the Cox proportional hazards model and is based on martingale residuals. In the GB test, the observations are divided into K groups according to their estimated risk score, an approach similar to Hosmer and Lemeshow goodness-of-fit for logistic regression^34^. Brier score was calculated at different time points, and a calibration plot was also used for assessing calibration. In a calibration plot, expected probabilities (predicted probabilities from the model) are plotted against observed outcome probabilities (calculated by Kaplan–Meier estimates). Arjas like plots were used for assessing the goodness of fit graphically^35^. We also produced histograms of the prognostic index (a linear predictor of the Cox model) to show the prognostic index distribution in the derivation and validation data set. We also assessed calibration using the approach proposed by Royston P^36^, where observed (Kaplan–Meier) and predicted survival probabilities compared in some prognostic groups derived by placing cut points on the prognostic index. We defined three risk groups (good, intermediate, and poor) from the 25th and 75th centiles of the prognostic index in the derivation dataset based on events.

We then created a point-based scoring system from the model so that it can be easily used in clinical practice. Integer points were assigned according to the presence/absence of each risk factor so that the overall risk can be estimated by summing the points together. We constructed the risk score utilizing the regression coefficients of our Cox model according to the method proposed by Sullivan et al.^37^. To facilitate calculating risk score, continuous variables considered in the model development were divided into categories as discussed before.

All statistical tests were two-sided. All statistical analyses were performed using Stata (Version 15.1; Stata Corporation, College Station, Texas 77845, USA).

### Comparing existing model performances to the developed model

We used a few existing hypertension risk models in our dataset to explain how our developed model performed in comparison to those models when those were applied to our population. Model selection was primarily made based on the availability of the final variables considered in those selected models in our dataset. This eliminated the majority of existing models from consideration for validation in our data set. In addition, whether the model provided enough information to perform the validation was also considered a major factor in selecting a model. For example, if a model did not provide regression coefficients or hazard or odds ratios from which coefficients can be derived were excluded from consideration. Also, if a model did not provide the predictive performance of their models, such as discrimination or calibration, they were excluded from considerations. In this case, we won’t be able to compare the validated model’s predictive performance in our dataset. Considering the aforementioned factors and information from our recent systematic review^38^, we selected the models by Parikh et al.^15^, Kivimӓki et al.^39^, Lim et al.^10^, Chien et al.^14^, and Wang et al.^12^ for validation in our dataset. The model by Parikh et al.^15^, also known as the Framingham Risk Score (FRS), was developed in the United States in a predominantly White population, with age, sex, systolic blood pressure (SBP), diastolic blood pressure (DBP), BMI, parental hypertension, cigarette smoking, and age by DBP as final variables. Kivimӓki et al.^39^ developed the Whitehall II Risk Score in England in a predominantly White population. In model construction, the same FRS variables were used. Lim et al.^10^ developed their model in Korea in an Asian population using the same variables as FRS. Chien et al.^14^ developed their model in Taiwan among the ethnic Chinese population. Two models were created, and we validated their clinical model using age, gender, BMI, SBP, and DBP as the final variables. Wang et al.^12^ developed their model in China with a rural Chinese population. The final variables in the model were age, parental hypertension, SBP, DBP, BMI, and age by BMI. The final variables considered in these models were available in our dataset.

This study’s ethics was approved by the Conjoint Health Research Ethics Board (CHREB) at the University of Calgary, and all methods were performed in accordance with the relevant guidelines and regulations. Informed consent was waived by the CHREB (REB18-0162_REN2) because the dataset used in this study consisted of de-identified secondary data released for research purposes.

### Patient consent

Not required. The manuscript is based on the analysis of secondary de-identified data. Patients and the public were not involved in the development, design, conduct or reporting of the study.

## Results

Baseline characteristics of the study participants are presented in Table 1 and supplementary table (Table S2). In Table 1, the study participants’ characteristics are given for the entire cohort as well as compared according to the status of developing hypertension. In contrast, in Table S2, characteristics are compared between the derivation sample and the validation sample. Overall, the study participants’ mean age was 50.99 years, and the participation of females (68.55%) in the study was higher than the males (31.45%). During the median 5.8-year follow-up, 625 (3.41%) participants developed hypertension. In Table 1, most of the study characteristics were significantly different (*p* < 0.05) between those who developed hypertension and those who did not. Those who developed hypertension were relatively older, had higher (average) BMI, DBP, SBP, and more with diabetes and cardiovascular disease. The proportions of males and females were also significantly different between these two groups. However, some study characteristics were similar with no statistically significant difference (*p* > 0.05), including ethnicity, family history of hypertension, alcohol consumption, and total physical activity time. When we randomly divided the data into derivation and validation sets (Table S2), the study characteristics were similar with no significant difference (*p* < 0.05) between the derivation and validation sample except BMI waist ratio.

**Table 1:** Baseline characteristics of study participants according to the status of developing hypertension or not.

Socio-demographic characteristics of groups					
Variable	Categories	All participants (18,322)	Participants who have developed hypertension (n = 625)	Participants who did not develop hypertension (n = 17,697)	*P* value
Age, years, mean (SE)		50.99 (0.07)	53.99 (0.35)	50.88 (0.07)	< 0.001
Age, years, n (%)	35 to less than 45	5556 (30.32)	107 (17.12)	5449 (30.79)	< 0.001
	45 to less than 55	6188 (33.77)	213 (34.08)	5975 (33.76)	
	55 to less than 65	5190 (28.33)	226 (36.16)	4964 (28.05)	
	≥ 65	1388 (7.58)	79 (12.64)	1309 (7.39)	
Sex, n (%)	Male	5763 (31.45)	250 (40)	5513 (31.15)	< 0.001
	Female	12,559 (68.55)	375 (60)	12,184 (68.85)	
Body mass index, kg/m2, mean (SE)		26.45 (0.04)	28.63 (0.21)	26.38 (0.04)	< 0.001
Body mass index, kg/m2, n (%)	Underweight (< 18.5)	179 (0.97)	–	–	< 0.001
	Normal (18.5–24.99)	7819 (42.68)	148 (23.62)	7642 (43.18)	
	Overweight (25.0–29.99)	6876 (37.53)	271 (43.37)	6501 (36.73)	
	Obese (≥ 30.0)	3448 (18.82)	203 (32.53)	3355 (18.96)	
BMI waist ratio, mean (SE)		0.28 (0.0002)	0.2893 (0.0013)	0.2831 (0.0002)	< 0.001
BMI waist ratio in quartiles, mean (SE)	Quartile 1	0.25 (0.0002)	0.25 (0.0009)	0.25 (0.0002)	0.485
	Quartile 2	0.27 (0.0001)	0.27 (0.0004)	0.27 (0.0001)	0.433
	Quartile 3	0.29 (0.0001)	0.29 (0.0005)	0.29 (0.0001)	0.118
	Quartile 4	0.32 (0.0003)	0.33 (0.0016)	0.32 (0.0003)	0.017
Hip circumference, mean (SE)		104.85 (0.08)	108.25 (0.44)	104.78 (0.08)	< 0.001
Waist circumference, mean (SE)		92.38 (0.10)	100.60 (0.60)	92.21 (0.10)	< 0.001
Waist circumference, n (%)	Normal (≤ 102 cm for male and ≤ 88 cm for female)	10,188 (55.60)	201 (32.11)	9987 (56.43)	< 0.001
	Substantially increased risk of metabolic complications (> 102 cm for male and > 88 cm for female)	8134 (44.40)	424 (67.89)	7710 (43.57)	
Waist hip ratio, mean (SE)		0.9093 (0.0006)	0.9363 (0.0033)	0.9085 (0.0006)	< 0.001
Waist hip ratio, n (%)	Normal (< 0.9 for male and < 0.85 for female)	4466 (24.38)	101 (16.08)	4366 (24.67)	< 0.001
	Abdominal obesity (≥ 0.9 for male and ≥ 0.85 for female)	13,856 (75.62)	524 (83.92)	13,331 (75.33)	
Body fat percentage, mean (SE)		31.86 (0.07)	34.67 (0.37)	31.84 (0.07)	< 0.001
Body fat percentage, n (%)	Normal (< 25.0 for male and < 35.0 for female)	9425 (51.44)	179 (28.59)	9246 (52.25)	< 0.001
	Obese (≥ 25.0 for male and ≥ 35.0 for female)	8897 (48.56)	446 (71.40)	8451 (47.75)	
Diastolic blood pressure, mean (SE)		72.96 (0.08)	78.43 (0.47)	72.78 (0.08)	< 0.001
Diastolic blood pressure, mmHg, n (%)	< 80	13,977 (76.28)	344 (55.05)	13,633 (77.03)	< 0.001
	80–89	3482 (19.00)	184 (29.44)	3298 (18.63)	
	≥ 90	863 (4.71)	97 (15.51)	766 (4.33)	
Systolic blood pressure, mean (SE)		119.71 (0.11)	132.36 (0.67)	119.40 (0.12)	< 0.001
Systolic blood pressure, mmHg, n (%)	< 120	9600 (52.40)	129 (20.69)	9471 (53.52)	< 0.001
	120–129	4585 (25.03)	139 (22.25)	4446 (25.12)	
	130–139	2684 (14.65)	176 (28.23)	2508 (14.17)	
	≥ 140	1453 (7.93)	180 (28.83)	1272 (7.19)	
Marital status, n (%)	Married and/or living with a partner	14,457 (78.91)	488 (78.08)	13,969 (78.94)	0.146
	Single, never married	1180 (6.44)	32 (5.12)	1148 (6.49)	
	Other (divorced, widowed, separated)	2685 (14.65)	105 (16.8)	2580 (14.57)	
Residence, n (%)	Urban	15,272 (83.35)	428 (68.48)	14,844 (83.88)	0.146
	Rural	3050 (16.65)	197 (31.52)	2853 (16.12)	
Total household income, n (%)	< $49,999	2800 (15.28)	178 (28.56)	2627 (14.84)	< 0.001
	$50,000–$99,999	5912 (32.27)	229 (36.68)	5690 (32.15)	
	$100,000–$199,999	7174 (39.16)	177 (28.27)	6986 (39.48)	
	≥ $200,000	2436 (13.29)	41 (6.49)	2394 (13.52)	
Highest education level completed, n (%)	High school or below (none, elementary school, high school, trade, technical or vocational school, apprenticeship training or technical CEGEP)	6164 (33.64)	309 (49.35)	5854 (33.08)	< 0.001
	Diploma but below bachelor’s degree (diploma from a community college, pre-university CEGEP or non-university certificate, university certificate below bachelor’s level)	4926 (26.89)	163 (26.15)	4764 (26.92)	
	Bachelor’s degree or above (bachelor's degree, graduate degree (MSc, MBA, MD, PhD, etc.))	7232 (39.47)	153 (24.49)	7079 (40.0)	
Ethnicity, n (%)	Aboriginal	68 (0.37)	–	–	0.349
	Asian (South Asian, East Asian, Southeast Asian, Filipino, West Asian, Arab)	827 (4.51)	21 (3.4)	806 (4.55)	
	White	16,894 (92.21)	588 (94.03)	16,307 (92.14)	
	Latin American Hispanic	162 (0.89)	–	–	
	Black	97 (0.53)	–	–	
	Other (Jewish and others)	273 (1.49)	11 (1.76)	262 (1.48)	
Diabetes, n (%)		735 (4.01)	58 (9.28)	677 (3.83)	< 0.001
Cardiovascular disease, n (%)		377 (2.06)	40 (6.4)	337 (1.9)	< 0.001
Depression, n (%)		2011 (10.98)	79 (12.64)	1932 (10.92)	0.179
Family history of hypertension, n (%)		10,946 (59.74)	396 (63.36)	10,550 (59.61)	0.061
Smoking status, n (%)	Never	10,107 (55.16)	290 (46.37)	9823 (55.51)	< 0.001
	Former	6773 (36.97)	276 (44.15)	6491 (36.68)	
	Current	1442 (7.87)	59 (9.48)	1383 (7.81)	
Ever smoked, n (%)		8215 (44.84)	335 (53.63)	7874 (44.49)	< 0.001
Alcohol consumption, n (%)	Never	1279 (6.98)	56 (8.97)	1224 (6.92)	0.189
	≤ 1 time a week	9642 (52.63)	341 (54.52)	9307 (52.59)	
	2 to 3 times a week	3820 (20.85)	123 (19.77)	3689 (20.85)	
	4 to 5 times a week	1988 (10.85)	55 (8.74)	1938 (10.95)	
	≥ 6 times a week	1593 (8.69)	50 (8.0)	1539 (8.69)	
Working status, n (%)	Full time	11,449 (62.49)	352 (56.29)	11,057 (62.48)	< 0.001
	Part time	4596 (25.09)	182 (29.19)	4422 (24.99)	
	Other (looking after home, disable/sick, student, unpaid/voluntary)	1857 (10.13)	83 (13.23)	1803 (10.18)	
	Unemployed	420 (2.29)	–	–	
Total sleep time, n (%)	≤ 5 h (short sleep duration)	1192 (6.51)	47 (7.49)	1147 (6.48)	< 0.001
	6 h	3732 (20.37)	127 (20.33)	3604 (20.37)	
	7 h (reference)	7048 (38.46)	200 (32.02)	6847 (38.69)	
	8 h	5115 (27.92)	185 (29.66)	4929 (27.85)	
	≥ 9 h (long sleep duration)	1235 (6.74)	66 (10.49)	1170 (6.61)	
Total physical activity time, mean (SE)		3159.83 (21.43)	3183.97 (126.52)	3157.58 (21.68)	0.825
Total physical activity time, n (%)	Light (< 450 MET minutes/week)	1668 (9.10)	84 (13.44)	1584 (8.95)	0.001
	Moderate (450–900 MET minutes/week)	2067 (11.28)	69 (11.04)	1998 (11.29)	
	Vigorous (> 900 MET minutes/week)	14,587 (79.61)	472 (75.52)	14,115 (79.76)	
Total sitting time, mean (SE)		2488.53 (8.92)	2389.16 (49.14)	2490.98 (9.38)	0.043
Physical activity, n (%)	Low (first quartile of physical activity time and fourth quartile of sitting time)	1685 (9.19)	59 (9.47)	1678 (9.48)	0.707
	Moderate (second and third quartile of physical activity time and sitting time)	14,478 (79.02)	488 (78.12)	13,957 (78.87)	
	High (fourth quartile of physical activity and first quartile of sitting time)	2159 (11.78)	78 (12.40)	2062 (11.65)	
Vegetable and fruit consumption, n (%)	Low consumption (less than 5 servings of vegetable and fruit)	15,264 (83.31)	544 (87.05)	14,721 (83.18)	0.024
	Moderate consumption (less than 5 servings of vegetable but more than 5 servings of fruit OR more than 5 servings of vegetable but less than 5 servings of fruits	2536 (13.84)	68 (10.84)	2469 (13.95)	
	High consumption (5 or more servings of vegetable and fruit)	522 (2.85)	13 (2.11)	507(2.87)	
Job schedule, n (%)	Regular daytime shift	12,866 (70.22)	385 (61.59)	12,452 (70.36)	< 0.001
	Other (evening shift, night shift, rotating shift, split shift, irregular shift, or on call)	5456 (29.78)	240 (38.41)	5245 (29.64)	

“–” indicates cell frequency < 10.

From the list of candidate variables, six (ever smoked, hip circumference, body fat percentage, BMI waist ratio, waist circumference, diastolic blood pressure.) were excluded from the model building due to their high collinearity (threshold VIF > 2.5) with other variables. Comparing the study characteristics between the missing and imputed is presented in the supplementary table (Table S3).

In the derivation sample, most of the candidate variables used in our study were identified as significant univariate predictors (Table 2). Variables not significantly associated with incident hypertension in univariate models were excluded from the multivariable model. In the multivariable model, age, sex, BMI, SBP, diabetes, CVD, total physical activity time, depression, waist-hip ratio, residence, highest education level completed, working status, total household income, family history of hypertension, smoking status, total sleep time, vegetable and fruit consumption, and job schedule was included. The multivariable Cox model indicated that age, BMI, SBP, diabetes, total physical activity time, and cardiovascular disease were independent risk factors of incident hypertension (Table 2). We forced sex into the model, considering its clinical importance. The following interaction terms were added to the model with other significant variables in the multivariable Cox model: age by BMI, age by SBP, age by diabetes, age by CVD, age by total physical activity time, age by sex, BMI by sex, SBP by sex, diabetes by sex, CVD by sex, and total physical activity time by sex. When the interaction terms were included in the model, age by sex, age by BMI, age by SBP, age by total physical activity time, sex by SBP, and sex by CVD showed significant association with incident hypertension (Table 3). However, the inclusion of these interaction terms did not improve the models’ discriminative performance. The models with and without interaction terms were virtually identical regarding their Harrel’s C-statistics value (0.77 and 0.77, respectively) and statistical significance (*p* = 0.64). Consequently, the interaction terms were excluded from the finally selected model. The model with only main effects was used in subsequent analyses to construct a simpler and more user-friendly risk estimation equation and risk score. A global test for Cox proportional hazards assumption indicated no violation of assumptions (*p* = 0.72) (Supplementary Table S4). The baseline survival function at median follow-up time 5.80-years ≈ 6-years, $S_{0} \left( 6 \right)$ was (0.977). In the derivation sample, the model’s discriminative performance (Harrel’s C-statistic) was 0.77.

**Table 2:** Unadjusted and adjusted hazard ratios for the risk factors of hypertension incidence.

Unadjusted and adjusted hazard ratios and 95% confidence intervals for the risk factors of hypertension incidence						
Variable	Unadjusted hazard ratio (95% CI)	*P* value		Adjusted hazard ratio (95% CI)	*P* value	
Age, years	1.05 (1.03–1.06)	< 0.001		1.02 (1.01–1.03)	0.002	
**Sex**						
Male	Reference			Reference		
Female	0.68 (0.56–0.82)	< 0.001		1.01 (0.80–1.28)	0.923	
Body mass index, kg/m^2^	1.07 (1.06–1.09)	< 0.001		1.05 (1.03–1.07)	< 0.001	
BMI waist ratio	1894.98 (93.43–38,435.67)	< 0.001				
Hip circumference, cm	1.03 (1.02–1.04)	< 0.001				
Waist circumference, cm	1.04 (1.03–1.05)	< 0.001				
Waist hip ratio	41.81 (12.45–140.43)	< 0.001		0.94 (0.22–4.04)	0.930	
Body fat percentage, percentage	1.03 (1.02–1.04)	< 0.001				
Diastolic blood pressure, mmHg	1.06 (1.05–1.07)	< 0.001				
Systolic blood pressure, mmHg	1.05 (1.05–1.06)	< 0.001		1.05 (1.04–1.05)	< 0.001	
**Marital status**						
Married or living with a partner	Reference		0.145*			
Single, never married	1.02 (0.66–1.58)	0.913				
Other (divorced, widowed, separated)	1.29 (1.00–1.66)	0.050				
**Residence**						
Urban	Reference			Reference		
Rural	1.37 (1.11–1.71)	0.004		1.08 (0.86–1.35)	0.500	
**Total household income**						
< $49,999	Reference		< 0.001*	Reference		0.060*
$50,000–$99,999	0.65 (0.51–0.83)	0.001		0.80 (0.62–1.04)	0.090	
$100,000–$199,999	0.51 (0.39–0.65)	< 0.001		0.75 (0.57–0.99)	0.048	
≥ $200,000	0.34 (0.22–0.52)	< 0.001		0.56 (0.36–0.88)	0.012	
**Highest education level completed**						
High school or below (none, elementary school, high school, trade, technical or vocational school, apprenticeship training or technical CEGEP)	Reference		< 0.001*	Reference		0.250*
Diploma but below bachelor's degree (diploma from a community college, pre-university CEGEP or non-university certificate, university certificate below bachelor's level)	0.79 (0.63–0.99)	0.050		1.01 (0.79–1.28)	0.952	
Bachelor’s degree or above (bachelor's degree, graduate degree (MSc, MBA, MD, PhD, etc.))	0.54 (0.43–0.69)	< 0.001		0.82 (0.63–1.06)	0.128	
**Ethnicity**						
Aboriginal	0.49 (0.07–3.50)	0.478	0.532*			
Asian (South Asian, East Asian, Southeast Asian, Filipino, West Asian, Arab)	1.17 (0.71–1.93)	0.543				
White	Reference					
Latin American Hispanic	0.33 (0.05–2.36)	0.270				
Black	0.62 (0.09–4.41)	0.632				
Other (Jewish and others)	1.61 (0.80–3.25)	0.182				
**Diabetes**						
No	Reference			Reference		
Yes	2.10 (1.48–2.98)	< 0.001		1.71 (1.19–2.46)	0.004	
**Cardiovascular disease**						
No	Reference			Reference		
Yes	3.14 (2.13–4.64)	< 0.001		2.81 (1.89–4.19)	< 0.001	
**Depression**						
No	Reference			Reference		
Yes	1.08 (0.79–1.46)	0.640		0.97 (0.71–1.33)	0.874	
**Family history of hypertension**						
No	Reference			Reference		
Yes	1.14 (0.93–1.39)	0.202		1.13 (0.93–1.39)	0.225	
**Smoking status**						
Never	Reference		0.031*	Reference		0.759*
Former	1.31 (1.07–1.61)	0.009		1.07 (0.87–1.32)	0.536	
Current	1.23 (0.87–1.74)	0.250		1.11 (0.78–1.58)	0.565	
**Ever smoked**						
No	Reference					
Yes	1.29 (1.07–1.57)	0.009				
**Alcohol consumption**						
Never	Reference		0.249*			
≤ 1 time a week	0.74 (0.53–1.04)	0.085				
2 to 3 times a week	0.86 (0.59–1.24)	0.414				
4 to 5 times a week	0.72 (0.47–1.10)	0.130				
≥ 6 times a week	0.63 (0.40–1.01)	0.058				
**Working status**						
Full time	Reference		< 0.001*	Reference		0.294*
Part time	0.89 (0.68–1.18)	0.426		0.83 (0.62–1.12)	0.232	
Other (looking after home, disable/sick, student, unpaid/voluntary)	1.63 (1.32–2.03)	< 0.001		0.96 (0.71–1.30)	0.807	
Unemployed	0.53 (0.20–1.41)	0.202		0.45 (0.16–1.23)	0.120	
**Total sleep time, hours**						
≤ 5 h (short sleep duration)	1.60 (1.11–2.31)	0.012	0.006*	1.03 (0.70–1.51)	0.882	0.178*
6 h	1.42 (1.08–1.85)	0.011		0.77 (0.53–1.12)	0.173	
7 h (reference)	Reference			Reference		
8 h	1.17 (0.91–1.51)	0.220		0.85 (0.59–1.24)	0.408	
≥ 9 h (long sleep duration)	1.70 (1.19–2.43)	0.003		1.07 (0.68–1.68)	0.781	
Total physical activity time, minutes/week	0.99 (0.99–1.00)	0.144		0.99 (0.99993–0.999997)	0.033	
Total sitting time, minutes/week	1.00 (0.99–1.01)	0.660				
**Physical activity, quartiles**						
Low (first quartile of physical activity time and fourth quartile of sitting time)	Reference		0.738*			
Moderate (second and third quartile of physical activity time and sitting time)	0.88 (0.64–1.21)	0.437				
High (fourth quartile of physical activity and first quartile of sitting time)	0.90 (0.60–1.35)	0.613				
**Vegetable and fruit consumption, servings**						
Low consumption (less than 5 servings of vegetable and fruit)	Reference		0.408*	Reference		0.494*
Moderate consumption (less than 5 servings of vegetable but more than 5 servings of fruit OR more than 5 servings of vegetable but less than 5 servings of fruits	0.81 (0.59–1.11)	0.191		0.97 (0.70–1.33)	0.832	
High consumption (5 or more servings of vegetable and fruit)	0.89 (0.48–1.67)	0.725		1.45 (0.77–2.74)	0.249	
**Job schedule**						
Regular daytime shift	Reference			Reference		
Other (evening shift, night shift, rotating shift, split shift, irregular shift, or on call)	1.42 (1.17–1.73)	< 0.001		1.15 (0.91–1.46)	0.229	

*Overall effect for categorical variables with multiple categories.

**Table 3:** Regression coefficients and hazard ratios for incident hypertension.

Variable	Simplified model without interaction terms				The model with interaction terms			
	β	Standard error (SE)	Hazard ratio (HR)	95% CI	β	Standard error (SE)	Hazard ratio (HR)	95% CI
Age	0.02768	0.00562	1.02807	1.02–1.04	0.18825	0.05158	1.20714	1.09–1.34
Sex*	0.08722	0.10411	1.09113	0.89–1.34	− 2.75995	1.02372	0.06329	0.01–0.47
Body mass index (BMI)	0.05147	0.00857	1.05282	1.04–1.07	0.13194	0.04638	1.14104	1.04–1.25
Systolic blood pressure (SBP)	0.04629	0.00309	1.04738	1.04–1.05	0.08233	0.01898	1.08581	1.05–1.13
Diabetes	0.57066	0.18200	1.76943	1.24–2.53	0.62335	0.18262	1.86517	1.30–2.67
Cardiovascular disease (CVD)	1.08710	0.20085	2.96566	2.00–4.39	1.43281	0.24367	4.19044	2.60–6.76
Total physical activity time	− 0.00003	0.00002	0.99997	0.99–1.00	0.00024	0.00010	1.00024	1.00–1.00
Age by sex					0.01516	0.01133	1.01527	0.99–1.04
Age by BMI					− 0.00157	0.00088	0.99843	0.99–1.00
Age by SBP					− 0.00084	0.00035	0.99916	0.99–0.99
Age by total physical activity time					− 0.00001	0.000002	0.99999	0.99–0.99
Sex by SBP					0.01583	0.00638	1.01596	1.00–1.03
Sex by CVD					− 0.96267	0.45499	0.38187	0.16–0.93

*Male is the reference category.

**Figure 1:**
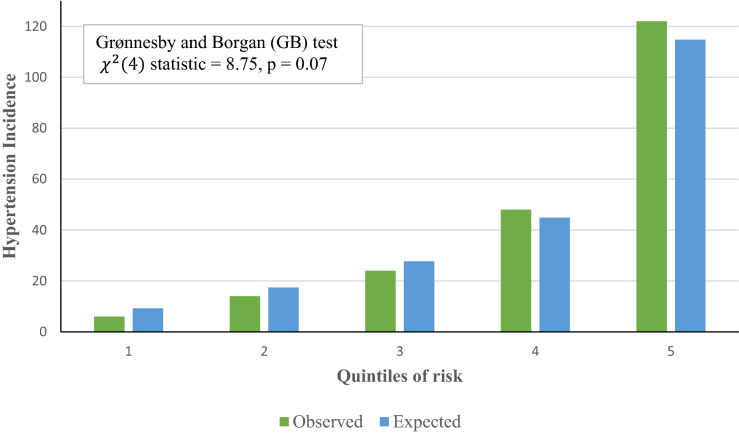
Grønnesby and Borgan (GB) goodness-of-fit test of the risk prediction model for incident hypertension in the validation sample.

**Figure 2:**
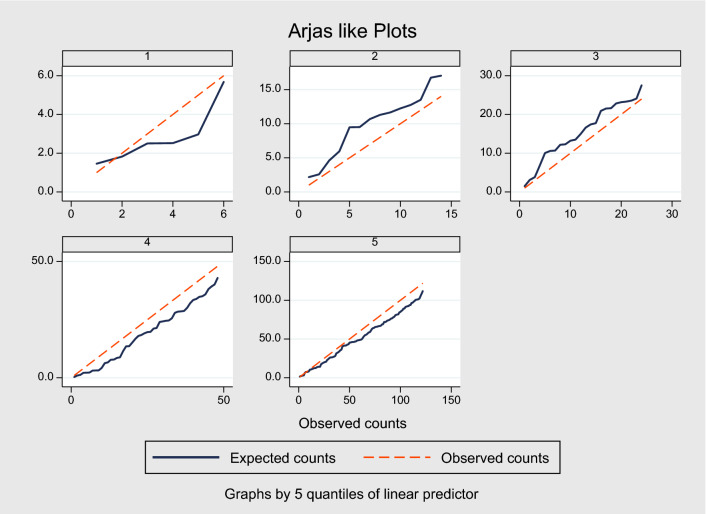
Arjas like plots to compare observed and expected events in five quantiles of the linear predictor in the validation sample.

**Figure 3:**
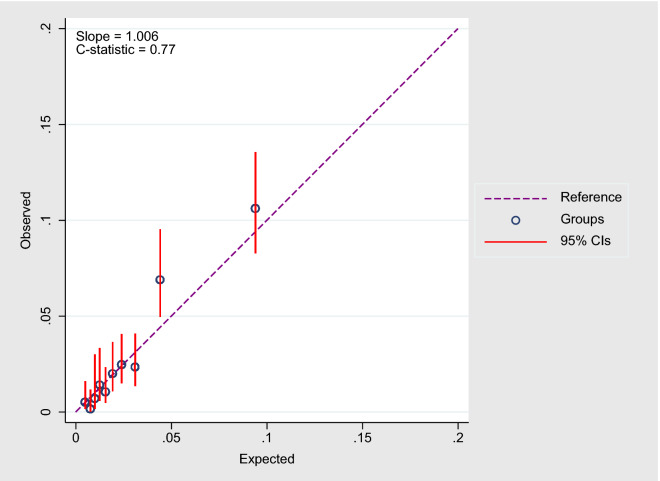
Calibration plot where expected probabilities (predicted probabilities from the model) are plotted against observed outcome probabilities (calculated by Kaplan–Meier estimates).

**Figure 4:**
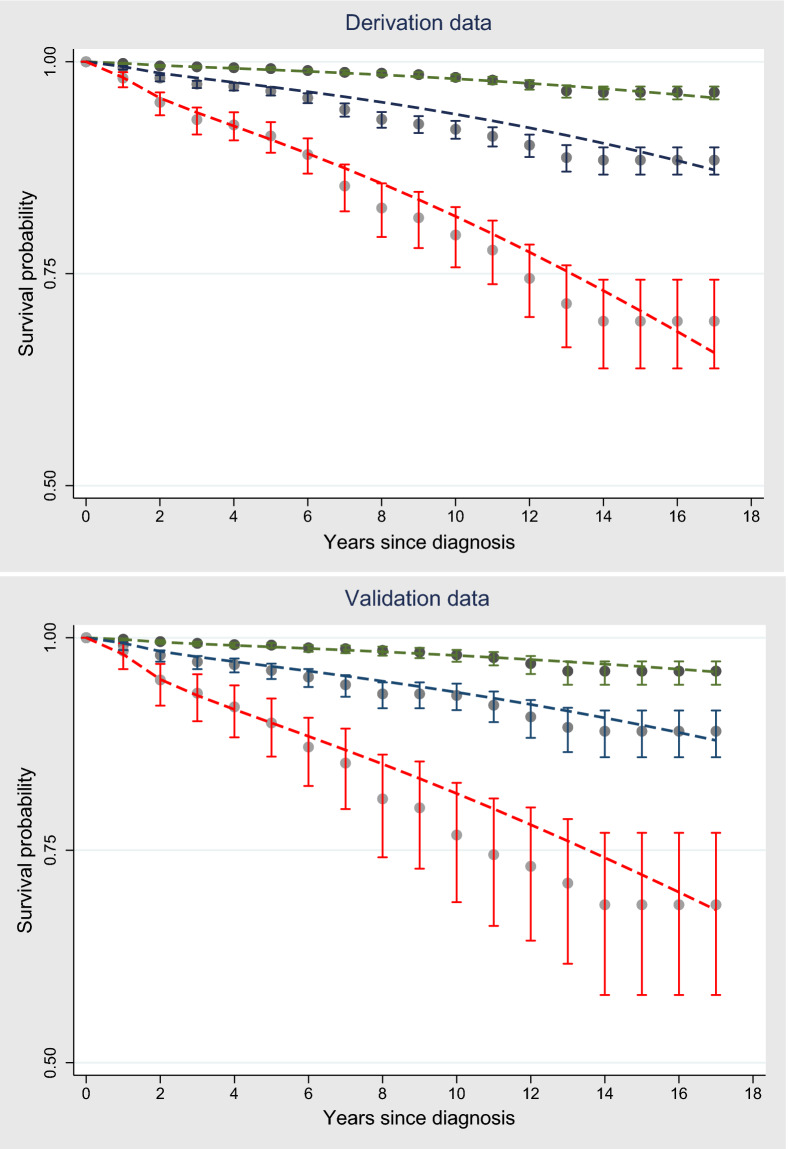
Smooth dashed lines represent predicted survival probabilities, and vertical capped lines represent Kaplan–Meier estimates with 95% confidence intervals. Three prognosis groups are plotted: the “Good” group (green lines), the “Intermediate” group (navy blue lines), and the “Poor” group (red lines).

**Figure 5:**
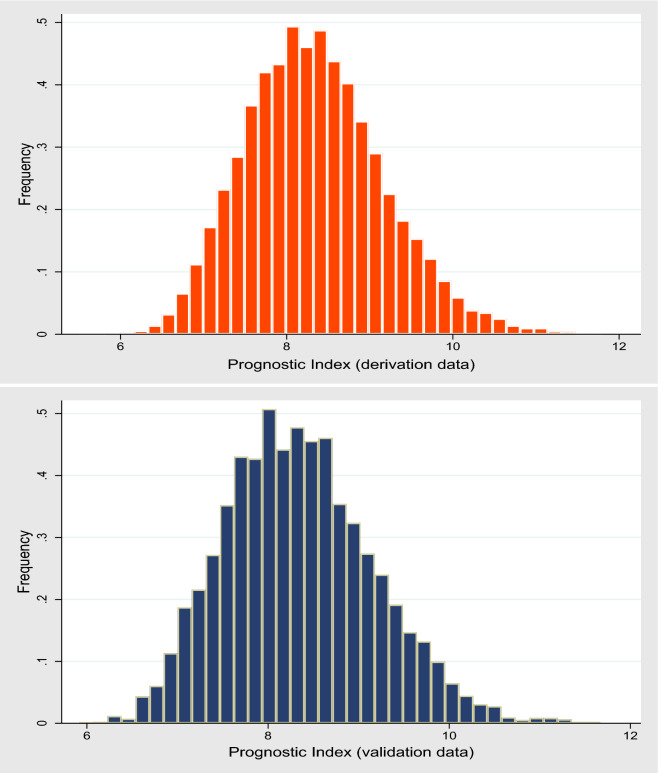
Histogram of the prognostic index in the derivation and validation datasets.

When we applied our derived model in the validation sample, the model’s discriminative performance was good (Harrel’s C-statistic 0.77). The results of the GB test indicated an acceptable calibration of the risk prediction model ($\chi^{2}$ statistic 8.75, *p* = 0.07, Fig. 1). To compare the observed and expected events in each group based on risk score, Arjas like plots are also presented (Fig. 2). A calibration plot of our prediction model at a time of 6-years was also presented in Fig. 3. A calibration slope of 1.006 indicates that predicted probabilities do not vary enough^40^. Figure 4 represents the calibration of our model in the derivation and validation datasets. The calibration of the model looks good in each dataset. The predictions in the validation dataset are good for both “Good” and “Intermediate” risk groups where survival and predicted probabilities are quite similar, except slightly higher predictions between 6- and 14-years time intervals for the “Intermediate” group. The predictions in the “Poor” group are consistent with the survival up to year six and somewhat high later; that is, survival tends to be worse than predicted. Due to fewer validation data events, the confidence intervals tend to be wider in validation data than in the derivation data. Figure 5 presents the prognostic index histogram in derivation and validation data, and no obvious irregularities and outliers were detected. Brier score calculated at 4-year, 5-year, 6-year, and 7-year time points are 0.018, 0.021, 0.026, and 0.029, respectively indicating accurate predictions.

Finally, from the developed model, a simple and practical risk score was created to calculate the risk of incident hypertension at different times (2-year, 3-year, 5-year, and 6-year) (Table 4). The constant for the points system or the number of regression units that will correspond to one point was set as the risk associated with a 5-year increase in age. To score a continuous variable, the range of possible values of the variable was divided into appropriate categories to enable the allocation of points to the selected categories. To determine the reference values for the open-ended categories (e.g., <or>), we used the 1st percentile and the 99th percentile of that variable to minimize the influence of extreme values. The points were initially computed as a decimal value, but later rounded to the nearest integer for facile calculation. The approximate risk of incident hypertension was then estimated via summation of the points awarded to each of the items. We attach the risks associated with each point total using the Cox regression equation (Table 5). Finally, we created risk categories according to the total points. In our model, the maximum total point is 40, and the minimum is − 2. For simple interpretation in a clinical setting, we categorize estimated risk into three categories and presented in Table 6.

**Table 4:** Calculation of point values for risk score

Variable	$\beta$	Categories	Reference value $\left( W \right)$	$\beta \left( {W - W_{REF} } \right)$	$Points$ $= \frac{{{\beta }\left( {W - W_{REF} } \right)}}{B}$
Age	0.02768	35 to less than 45 *	39.5 $(W_{REF} )$	0	0
		45 to less than 55	49.5	0.2768	2
		55 to less than 65	59.5	0.5536	4
		65 to less than 75	69.5	0.8304	6
Sex	0.08722	Male *	0 $(W_{REF} )$	0	0
		Female	1	0.0872	1
Body mass index^a^	0.05147	< 18.5 *	18.5 $\left( {W_{REF} } \right)$	0	0
		18.5 to less than 25.0	21.75	0.1673	1
		25.0 to less than 30.0	27.5	0.4632	3
		≥ 30.0	36.35	0.9187	7
Systolic blood pressure^b^	0.04629	< 120 *	106 $\left( {W_{REF} } \right)$	0	0
		120 to less than 130	125	0.8795	6
		130 to less than 140	135	1.3424	10
		≥ 140	148	1.9442	14
Diabetes	0.57066	No *	0 $\left( {W_{REF} } \right)$	0	0
		Yes	1	0.5707	4
Cardiovascular disease	1.08710	No *	0 $(W_{REF} )$	0	0
		Yes	1	1.0871	8
Physical activity total**	− 0.00003	Light (< 450 MET minutes/week)	274.5 $\left( {W_{REF} } \right)$	0	0
		Moderate (450–900 MET minutes/week)	675	− 0.0120	− 1
		Vigorous (> 900 MET minutes/week)	7209	− 0.2080	− 2

*Reference category.

The age range in the sample is 35–70.

^a^The range of body mass index is 12.5–64.9. To determine the reference values for the first and last categories, we use the 1st percentile (18.5) and the 99th percentile (42.7) to minimize extreme values’ influence.

**The range of physical activity total is from 33 MET minutes/week to 19,278 MET minutes/week. To determine the reference values for the first and last categories, we use the 1st percentile (99) and the 99th percentile (13,518) to minimize extreme values’ influence.

^b^The range of systolic blood pressures is 76–205. To determine the reference values for the first and last categories, we use the 1st percentile (92) and the 99th percentile (156) to minimize extreme values’ influence.

The constant for the points system or the number of regression units will correspond to one point. Here, we let *B* reflect the increase in risk associated with a 5-year increase in age:

$$ B = 5\left( {0.02768} \right) = 0.1384$$.

**Table 5:** Risk estimates for point totals at 2, 3, 5, and 6-year time.

2-year risk (%)		3-year risk (%)		5-year risk (%)		6-year risk (%)	
Point total	Estimate of risk	Point total	Estimate of risk	Point total	Estimate of risk	Point total	Estimate of risk
− 2	0.27	− 2	0.30	− 2	0.39	− 2	0.48
− 1	0.31	− 1	0.35	− 1	0.45	− 1	0.55
0	0.35	0	0.40	0	0.52	0	0.63
1	0.40	1	0.46	1	0.60	1	0.72
2	0.46	2	0.53	2	0.68	2	0.83
3	0.53	3	0.61	3	0.79	3	0.95
4	0.61	4	0.70	4	0.90	4	1.09
5	0.70	5	0.80	5	1.04	5	1.25
6	0.81	6	0.92	6	1.19	6	1.43
7	0.93	7	1.05	7	1.36	7	1.64
8	1.06	8	1.21	8	1.56	8	1.88
9	1.22	9	1.38	9	1.79	9	2.16
10	1.40	10	1.59	10	2.06	10	2.48
11	1.60	11	1.82	11	2.36	11	2.84
12	1.84	12	2.09	12	2.71	12	3.25
13	2.11	13	2.40	13	3.10	13	3.73
14	2.42	14	2.75	14	3.55	14	4.27
15	2.77	15	3.15	15	4.07	15	4.89
16	3.18	16	3.61	16	4.66	16	5.59
17	3.64	17	4.13	17	5.33	17	6.40
18	4.17	18	4.73	18	6.10	18	7.31
19	4.78	19	5.41	19	6.97	19	8.35
20	5.47	20	6.19	20	7.96	20	9.53
21	6.25	21	7.08	21	9.09	21	10.86
22	7.15	22	8.08	22	10.37	22	12.37
23	8.16	23	9.23	23	11.81	23	14.07
24	9.32	24	10.52	24	13.44	24	15.98
25	10.62	25	11.98	25	15.28	25	18.13
26	12.10	26	13.64	26	17.34	26	20.52
27	13.77	27	15.50	27	19.64	27	23.19
28	15.64	28	17.58	28	22.21	28	26.14
29	17.74	29	19.91	29	25.05	29	29.39
30	20.10	30	22.51	30	28.19	30	32.94
31	22.71	31	25.39	31	31.64	31	36.80
32	25.61	32	28.56	32	35.39	32	40.96
33	28.81	33	32.04	33	39.45	33	45.41
34	32.31	34	35.83	34	43.79	34	50.10
35	36.12	35	39.92	35	48.40	35	54.99
36	40.23	36	44.29	36	53.23	36	60.02
37	44.63	37	48.93	37	58.22	37	65.10
38	49.28	38	53.78	38	63.29	38	70.15
39	54.14	39	58.78	39	68.37	39	75.06
40	59.15	40	63.86	40	73.33	40	79.70

We determine the risks that are associated with each point in total.

The first step is to select the point totals’ theoretical range based on the point system computed earlier.

In our point system, the theoretical range of point totals is − 2 to 40.

We then attached a risk estimate to each point total using the Cox regression equation.

**Table 6:** Risk categories based on total points.

Total score	Risk category (based on 5-years estimated risk)
< 22 (< 10% estimated risk)	Low risk
22–27 (10–20% estimated risk)	Intermediate risk
> 27 (> 20% estimated risk)	High risk

### Case study

A 50-year-old male with BMI 28.5, SBP 135, diabetic, no CVD, and moderate physical activity (850 MET minutes/week).

**Table:** 

Risk factor	Value	Points
Age	50	2
Sex	Male	0
BMI	28.5	3
SBP	135	10
Diabetes status	Yes	4
CVD status	No	0
Physical activity	Moderate (850 MET minutes/week)	− 1
Point total		18
The estimate of risk (6-year)		7.31

The risk estimate based on our newly developed Cox model is computed as follows:

$$ \begin{aligned} \mathop \sum \limits_{i = 1}^{7} \beta_{i} X_{i} & = 0.02768\left( {50} \right) + 0.08722\left( 0 \right) + 0.05147\left( {28.5} \right) + 0.04629\left( {135} \right) \\ & \quad + 0.57066\left( 1 \right) + 1.08710\left( 0 \right) - 0.00003\left( {850} \right) = 9.645205 \\ \mathop \sum \limits_{i = 1}^{7} \beta_{i} \overline{X}_{i} & = 0.02768\left( {50.94} \right) + 0.08722\left( {0.3142} \right) + 0.05147\left( {26.48} \right) + 0.04629\left( {119.75} \right) \\ & \quad + 0.57066\left( {0.041} \right) + 1.08710\left( {0.021} \right) - 0.00003\left( {3157.97} \right) = 8.2950638 \\ \hat{p} & = 1 - S_{0} \left( t \right)^{{\exp \left( {\mathop \sum \limits_{i = 1}^{7} \beta_{i} X_{i} - \mathop \sum \limits_{i = 1}^{7} \beta_{i} \overline{X}_{i} } \right)}} = 1 - 0.977^{{\exp \left( {9.645205 - 8.2950638} \right)}} = 0.085 \\ \end{aligned} $$

The points system gives a 6-year risk of incident hypertension of 7.3%, while employing the Cox model directly gives an estimate of 8.5%.

### Existing models' performances in our dataset

**Figure 6:**
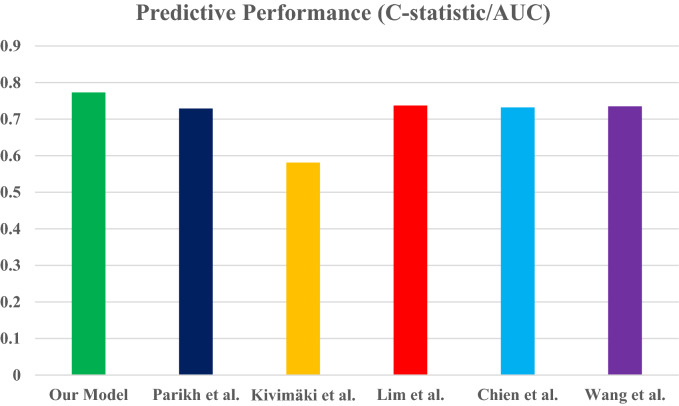
Comparison of the newly developed model's prediction performance with that of some previously published models.

We compared the models’ predictive performance using the most commonly reported predictive performance metric, the C-statistic. Table 7 shows the C-statistics from the original model and the C-statistics when the models were applied to our dataset. All models’ performances were lower in our population than their original predictive performance. Figure 6 compares our model’s predictive performance (C-statistic) to the five validated models. Our model’s better predictive performance was observed, which supports the creation of our new prediction model for the Canadian population.

**Table 7:** The predictive performance of some of the past published hypertension prediction models in our dataset.

Model	Original predictive performance (C-statistic/AUC)	Predictive performance in our dataset (C-statistic/AUC)
Parikh et al.	0.788	0.729
Kivimӓki et al.	0.804	0.581
Lim et al.	0.791	0.737
Chien et al.	0.737	0.732
Wang et al.	0.791	0.735

## Discussion

In this large prospective cohort study, we developed a simple model to predict the risk of developing hypertension incidence in Canadian adults. The variables included in our model (age, sex, SBP, BMI, diabetes, cardiovascular disease, and self-reported total physical activity time) are routinely and easily assessed in the primary-care clinical setting. Our prediction model for hypertension risk had very good discrimination and calibration for both the derivation and validation samples, suggesting that this model has good performance and may perform well when applied to a different Canadian population. Also, a risk score table was derived for clinical implementation and workability of the developed model. Derived point-based score where points assigned to each variable is easy to administer by health care professionals and the general population and can guide clinical counseling and decision making.

The predictive performance of our model was similar to other studies. Although prediction models’ performance varies considerably across studies, our recent meta-analysis on the predictive performance of hypertension risk prediction models indicates an overall pooled C-statistic of 0.75 [95% CI: 0.73–0.77]^41^, which justifies our model’s good predictive performance. Framingham hypertension risk score^15^, the most validated hypertension risk prediction model, had a C-statistic of 0.78, similar to our model. Our model’s calibration was also right on several performance measures.

Most of the variables included in our final model are consistent with other previous studies (Supplementary Fig. S1). The variable sex was not identified as a significant factor in our model, but we forced it into the model considering its clinical implication^42^. Diabetes and CVD were the two significant risk factors in our model, often excluded by many studies. Individuals who have diabetes or CVD have a higher risk of developing hypertension than those free of these conditions. Our risk prediction model aimed to identify the risk factors for hypertension in adults but excluding people with diabetes and CVD would limit our results’ generalizability. To develop a risk prediction model applicable to as many individuals as possible, we considered diabetes and CVD subjects in model building. Smoking, alcohol consumption, and family history of hypertension are common risk factors used in the past hypertension risk prediction models (Supplementary Fig. S1). In our study, these risk factors were not identified as significant. Their inclusion in the model also did not change the model’s discriminative performance (Harrel’s C-statistic remains the same as 0.77). We identified total physical activity time significantly contributes to our model. This finding is significant because exercise is considered a preventive factor for hypertension incidence supported by scientific evidence^43^. Moreover, it is a highly modifiable lifestyle factor, and physical activity changes can modify the status of hypertension incidence.

We assessed interaction effects in our model, and several of the interaction terms were identified as significant. However, inclusion of interaction terms in the model did not improve the model’s predictive performance. Our focus was on generating a simple and user-friendly risk scoring algorithm avoiding complexity. As a result, the interaction terms were excluded from the model in final considerations.

To our knowledge, this is the first hypertension risk prediction model developed explicitly in a Canadian population. The model was created using a large sample size, and the estimates from our prediction models were found to be stable, as demonstrated in the internal validation. Further, consideration of many candidate variables in model building is also a strength of this study. In contrast to most studies, where models were developed in complete cases, excluding those with missing values, we imputed missing values in our study. This approach prevented information loss, maximized information utilization, and made the results robust.

We could have used an existing model and evaluated its performance through external validation in our dataset before creating a new risk score. However, we refrain from doing this for the following reasons: First, when applied to new individuals, a prediction model typically performs worse than it did with its original study population^18,44^. When a low predictive accuracy is discovered after an external validation study, researchers must decide whether to reject the model or update it to improve its predictive accuracy. By combining information captured in the original model with information from new individuals from the validation study, the model can be updated or recalibrated for local circumstances^18^. Model updating entails adding more predictors or altering a portion of the formula to better suit the external population^18^. The appropriateness of model updating during external validation is a point of contention among researchers. Some claim that the researchers are developing a new prediction model even with minor changes^18,45,46^. Second, developing a new prediction model along with externally validating a well-known existing prediction model in the development cohort and concluding that the new model performs better is an inappropriate comparison in our view. Because this is then comparing the performance of one model in development to the performance of another model in external validation^18^. The newly developed model will almost always appear superior because it is optimally designed to fit the development data^18^. The performance of two existing prediction models should be directly compared in an external validation dataset that is independent of both model development cohorts. Given this, we did not evaluate an existing model's performance and then develop a new model on the same dataset. Nevertheless, for the purpose of comparison, we assessed a few of the published hypertension risk prediction models in our population and found that their performance was inferior to ours.

Our study has several limitations. Study participants were middle-aged Canadians. Prevention strategies are likely to be more effective if the young population can be targeted. Nevertheless, our study participants’ age range will likely have minimal impact on our study’s generalizability, as essential hypertension develops in the middle aged adults^47^, as represented here. At baseline, we excluded participants with self-reported hypertension, which can potentially lead to misclassification of hypertension status. The incidence rate of hypertension in our study was relatively low compared to what is reported for the general Alberta population^48^. There can be several potential reasons for that. The characteristics of the study participants in ATP may be different from the general Alberta population. For example, female participation in ATP data was more than double the male participation (69% vs. 31%), and the hypertension incidence rate in Alberta was much lower in females than the males in study age groups^48^. A potential selection bias also may lead to a lower incidence rate of hypertension in our study The participants in ATP were mainly selected using the volunteer sampling method^49^. Those who decided to join the study (i.e., who self-select into the survey) may have a different characteristic (e.g., healthier) than the non-participants. Due to the longitudinal nature of the study, there can also be a loss of study participants during follow-up. Participants who were lost to follow-up (e.g., due to emigration out of the province) may be more likely to develop hypertension. Our study ascertained outcome hypertension from a linked administrative health data (the hospital discharge abstract or physician claims data source) due to a lack of longitudinal data in ATP. There is a possibility that the outcome ascertainment was incomplete as we did not have measured blood pressure to verify. Also, people who did not have a healthcare encounter after cohort enrollment (e.g., did not visit a family physician/general practitioner or were not admitted to the hospital during the study period) were missed and can potentially lead to a lower hypertension incidence. We did not account for competing risks in our study because the expected event (death) rate is low as the cohort was healthy and relatively young at inception with a short follow-up time. We did not include genetic risk factors or biomarkers in our model. The inclusion of genetic risk factors in the model has the potential of improving risk prediction. However, our recent meta-analysis on hypertension risk prediction models^41^ and previous studies^11^ did not show any differences in discriminative performance (pooled C-statistic was 0.76 for models developed using genetic risk factors/biomarkers). In addition, the inclusion of genetic risk factors in the model may decrease the prediction model’s application in routine clinical practice. Sodium intake is an important dietary factor for the risk of incident hypertension; however, in our study, sodium intake data were not available. We could not perform an external validation of our model, essential for any prediction model’s generalizability. Therefore, further validation of our model in other populations, particularly in another Canadian jurisdiction, is warranted. A direct comparison of our models' performance with other models on the same dataset will allow us to properly understand the quality of our new model, allowing for a head-to-head comparison of predictive performance between models. The direct comparison will show which model performs best, which will help guide future research and clinical practice. In the future, we plan to compare our model to other relevant models on a separate dataset.

In conclusion, we have developed a simple yet practical prediction model to estimate the risk of incident hypertension for the Canadian population. Risk assessment tools are believed to be convenient in motivating high-risk individuals for future health problems to modify their lifestyles to decrease their risks. Once the model is validated via external validation studies, it can help identify individuals at higher risk of hypertension, increase health consciousness, motivate individuals to improve their lifestyles and prevent or delay the onset of hypertension.

## Supplementary Information

Supplementary Information.

## Data Availability

The data that support the findings of this study are available from Alberta’s Tomorrow Project (ATP) but restrictions apply to the availability of these data, which were used under license for the current study, and so are not publicly available. Data are however available from the authors upon reasonable request and with permission of Alberta’s Tomorrow Project (ATP).

## References

[1] Leung AA, Williams JVA, McAlister FA, Campbell NRC, Padwal RS (2020). Worsening hypertension awareness, treatment, and control rates in Canadian women between 2007 and 2017. Can. J. Cardiol..

[2] Bromfield S, Muntner P (2013). High blood pressure: The leading global burden of disease risk factor and the need for worldwide prevention programs. Curr. Hypertens. Rep..

[3] Nerenberg KA, Zarnke KB, Leung AA (2018). Hypertension Canada’s 2018 guidelines for diagnosis, risk assessment, prevention, and treatment of hypertension in adults and children. Can. J. Cardiol..

[4] Leung AA, Bushnik T, Hennessy D, McAlister FA, Manuel DG (2019). Risk factors for hypertension in Canada. Heal Rep..

[5] Chowdhury MZI, Turin TC (2020). Precision health through prediction modelling: Factors to consider before implementing a prediction model in clinical practice. J. Prim. Health Care.

[6] Chowdhury MZI, Turin TC (2021). Validating prediction models for use in clinical practice: Concept, steps, and procedures focusing on hypertension risk prediction. Hypertens. J..

[7] Chowdhury MZI, Yeasmin F, Rabi DM, Ronksley PE, Turin TC (2019). Predicting the risk of stroke among patients with type 2 diabetes: A systematic review and meta-analysis of C-statistics. BMJ Open.

[8] Kanegae H, Oikawa T, Suzuki K, Okawara Y, Kario K (2018). Developing and validating a new precise risk-prediction model for new-onset hypertension: The Jichi Genki hypertension prediction model (JG model). J. Clin. Hypertens..

[9] Otsuka T, Kachi Y, Takada H (2015). Development of a risk prediction model for incident hypertension in a working-age Japanese male population. Hypertens. Res..

[10] Lim NK, Son KH, Lee KS, Park HY, Cho MC (2013). Predicting the risk of incident hypertension in a Korean middle-aged population: Korean genome and epidemiology study. J. Clin. Hypertens..

[11] Paynter NP, Cook NR, Everett BM, Sesso HD, Buring JE, Ridker PM (2009). Prediction of incident hypertension risk in women with currently normal blood pressure. Am. J. Med..

[12] Wang B, Liu Y, Sun X (2020). Prediction model and assessment of probability of incident hypertension: The Rural Chinese cohort study. J. Hum. Hypertens..

[13] Kadomatsu Y, Tsukamoto M, Sasakabe T (2019). A risk score predicting new incidence of hypertension in Japan. J. Hum. Hypertens..

[14] Chien KL, Hsu HC, Su TC (2011). Prediction models for the risk of new-onset hypertension in ethnic Chinese in Taiwan. J. Hum. Hypertens..

[15] Parikh NI, Pencina MJ, Wang TJ (2008). A risk score for predicting near-term incidence of hypertension: The Framingham heart study. Ann. Intern Med..

[16] Giampaoli S, Palmieri L, Mattiello A, Panico S (2005). Definition of high risk individuals to optimise strategies for primary prevention of cardiovascular diseases. Nutr. Metab. Cardiovasc. Dis..

[17] Chowdhury MZI, Yeasmin F, Rabi DM, Ronksley PE, Turin TC (2019). Prognostic tools for cardiovascular disease in patients with type 2 diabetes: A systematic review and meta-analysis of C-statistics. J. Diabetes Complicat..

[18] Ramspek CL, Jager KJ, Dekker FW, Zoccali C, van Diepen M (2021). External validation of prognostic models: What, why, how, when and where?. Clin. Kidney J..

[19] Riley RD, Ensor J, Snell KIE (2016). External validation of clinical prediction models using big datasets from e-health records or IPD meta-analysis: Opportunities and challenges. BMJ.

[20] Moons KGM, Altman DG, Reitsma JB (2015). Transparent reporting of a multivariable prediction model for individual prognosis or diagnosis (TRIPOD): Explanation and elaboration. Ann. Intern. Med..

[21] Altman DG, Vergouwe Y, Royston P, Moons KGM (2009). Prognosis and prognostic research: Validating a prognostic model. BMJ.

[22] Altman DG, Royston P (2000). What do we mean by validating a prognostic model?. Stat. Med..

[23] Robson PJ, Solbak NM, Haig TR (2016). Design, methods and demographics from phase I of Alberta’s Tomorrow Project cohort: A prospective cohort profile. C Open.

[24] Sun D, Liu J, Xiao L (2017). Recent development of risk-prediction models for incident hypertension: An updated systematic review. PLoS ONE.

[25] Echouffo-Tcheugui JB, Batty GD, Kivimäki M, Kengne AP (2013). Risk models to predict hypertension: A systematic review. PLoS ONE.

[26] Quan H, Khan N, Hemmelgarn BR (2009). Validation of a case definition to define hypertension using administrative data. Hypertension.

[27] Kang H (2013). The prevention and handling of the missing data. Korean J. Anesthesiol..

[28] Sinharay S, Stern HS, Russell D (2001). The use of multiple imputation for the analysis of missing data. Psychol. Methods.

[29] Royston P, White IR (2011). Multiple imputation by chained equations (MICE): Implementation in stata. J. Stat. Softw..

[30] Midi H, Sarkar SK, Rana S (2010). Collinearity diagnostics of binary logistic regression model. J. Interdiscip. Math..

[31] Chowdhury MZI, Turin TC (2020). Variable selection strategies and its importance in clinical prediction modelling. Fam. Med. Community Heal.

[32] Harrell FE, Califf RM, Pryor DB, Lee KL, Rosati RA (1982). Evaluating the yield of medical tests. JAMA J. Am. Med. Assoc..

[33] Grønnesby JK, Borgan Ø (1996). A method for checking regression models in survival analysis based on the risk score. Lifetime Data Anal..

[34] Hosmer DW, Lemeshow S (1980). Goodness of fit tests for the multiple logistic regression model. Commun. Stat. Theory Methods.

[35] Arjas E (1988). A graphical method for assessing goodness of fit in Cox’s proportional hazards model. J. Am. Stat. Assoc..

[36] Royston P (2015). Tools for checking calibration of a Cox model in external validation: Prediction of population-averaged survival curves based on risk groups. Stata J..

[37] Sullivan LM, Massaro JM, D’Agostino RB (2004). Presentation of multivariate data for clinical use: The Framingham Study risk score functions. Stat. Med..

[38] Chowdhury MZI, Naeem I, Quan H, Palazón-Bru A (2022). Prediction of hypertension using traditional regression and machine learning models: A systematic review and meta-analysis. PLoS ONE.

[39] Kivimäki M, Batty GD, Singh-Manoux A (2009). Validating the Framingham hypertension risk score: Results from the Whitehall II study. Hypertension.

[40] Stevens RJ, Poppe KK (2020). Validation of clinical prediction models: What does the “calibration slope” really measure?. J. Clin. Epidemiol..

[41] Chowdhury, M. Z. I. Develop a comprehensive hypertension prediction model and risk score in population-based data applying conventional statistical and machine learning approaches. Published online 2021. 10.11575/PRISM/38706.

[42] Ramirez LA, Sullivan JC (2018). Sex differences in hypertension: Where we have been and where we are going. Am. J. Hypertens..

[43] Kshirsagar AV, Chiu YL, Bomback AS (2010). A hypertension risk score for middle-aged and older adults. J. Clin. Hypertens..

[44] Chowdhury MZI, Naeem I, Quan H (2020). Summarising and synthesising regression coefficients through systematic review and meta-analysis for improving hypertension prediction using metamodelling: Protocol. BMJ Open.

[45] Riley RD, Snell KIE, Ensor J (2019). Minimum sample size for developing a multivariable prediction model: PART II—Binary and time-to-event outcomes. Stat. Med..

[46] Moons KGM, Kengne AP, Grobbee DE (2012). Risk prediction models: II. External validation, model updating, and impact assessment. Heart.

[47] Hajjar I, Kotchen TA (2003). Trends in prevalence, awareness, treatment, and control of hypertension in the United States, 1988–2000. J. Am. Med. Assoc..

[48] *Interactive Health Data Application—Display Results*. Accessed March 29, 2021. http://www.ahw.gov.ab.ca/IHDA_Retrieval/selectSubCategoryParameters.do.

[49] Ye M, Robson PJ, Eurich DT, Vena JE, Xu JY, Johnson JA (2017). Cohort profile: Alberta’s Tomorrow Project. Int. J. Epidemiol..

