# Supplementary material for: Development and validation of a hypertension risk prediction model and construction of a risk score in a Canadian population
Source: Sci Rep. 2022 Jul 27;12:12780. doi: 10.1038/s41598-022-16904-x (PMC9329335; doi:10.1038/s41598-022-16904-x)
Supplement: Supplementary file 1 — Supplementary Information. [file 41598_2022_16904_MOESM1_ESM.doc]

**SUPPLEMENTARY MATERIALS**

**SUPPLEMENTARY FIGURE LEGENDS**

**Figure S1.** Traditional risk factors considered by conventional regression-based models.

**Figure S1.**


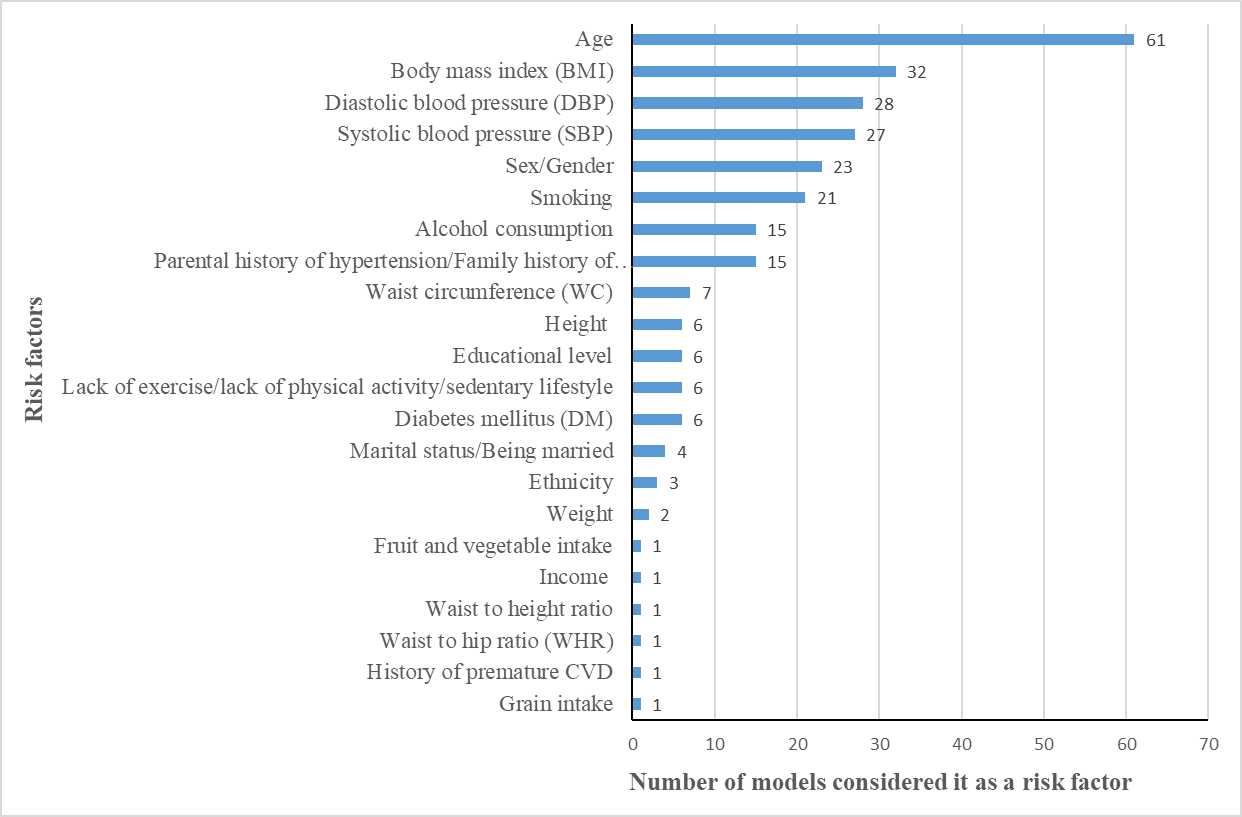


**Table S1.** Missing information about different variables

| **Variables** | **Missing** | **Total** | **Percent Missing** |
| --- | --- | --- | --- |
| Total Physical Activity Time | 520 | 18,322 | 2.84 |
| Total Sitting Time | 1,421 | 18,322 | 7.76 |
| Depression | 16 | 18,322 | 0.09 |
| Diabetes | — | 18,322 | — |
| Waist Hip Ratio | 4,686 | 18,322 | 25.58 |
| Sex | 0 | 18,322 | 0.00 |
| Age | 0 | 18,322 | 0.00 |
| Residence | 0 | 18,322 | 0.00 |
| Family History of Hypertension | 0 | 18,322 | 0.00 |
| Diastolic Blood Pressure | 4,283 | 18,322 | 23.38 |
| Systolic Blood Pressure | 4,283 | 18,322 | 23.38 |
| Ethnicity | 23 | 18,322 | 0.13 |
| Cardiovascular Disease | 0 | 18,322 | 0.00 |
| Highest Education Level Completed | 11 | 18,322 | 0.06 |
| Working Status | 0 | 18,322 | 0.00 |
| Vegetable and Fruit Consumption | 266 | 18,322 | 1.45 |
| Physical Activity | 1,846 | 18,322 | 10.08 |
| Total Household Income | 1,402 | 18,322 | 7.65 |
| Alcohol Consumption | 846 | 18,322 | 4.62 |
| Total Sleep Time | 239 | 18,322 | 1.30 |
| Smoking Status | 45 | 18,322 | 0.25 |
| Job Schedule | 4,303 | 18,322 | 23.49 |
| Marital Status | — | 18,322 | — |
| Body Mass Index | 4,260 | 18,322 | 23.25 |
| BMI Waist Ratio | 4,718 | 18,322 | 25.75 |
| Ever Smoked | 41 | 18,322 | 0.22 |
| Body Fat Percentage | 4,471 | 18,322 | 24.40 |
| Hip Circumference | 4,564 | 18,322 | 24.91 |
| Waist Circumference | 4,769 | 18,322 | 26.03 |

“—" indicates cell frequency < 10.

**Table S2.** Baseline characteristics of study participants and comparison in the derivation sample and validation sample

| **Socio-demographic characteristics of groups** | | | | | |
| --- | --- | --- | --- | --- | --- |
| **Variable** | **Categories** | **All participants**  **(18,322)** | **Derivation sample**  **(n =** **12,233)** | **Validation sample**  **(n =** **6,089)** | **P-value** |
| Age, years, mean (SE) |  | 50.99 (9.20) | 50.94 (9.19) | 51.07 (9.24) | 0.377 |
| Age, years, n (%) | 35 to less than 45 | 5,556 (30.32) | 3,723 (30.43) | 1,833 (30.10) | 0.275 |
|  | 45 to less than 55 | 6,188 (33.77) | 4,169 (34.08) | 2,019 (33.16) |  |
|  | 55 to less than 65 | 5,190 (28.33) | 3,410 (27.88) | 1,780 (29.23) |  |
|  | ≥ 65 | 1,388 (7.58) | 931 (7.61) | 457 (7.51) |  |
| Sex, n (%) | Male | 5,763 (31.45) | 3,844 (31.42) | 1,919 (31.52) | 0.899 |
|  | Female | 12,559 (68.55) | 8,389 (68.58) | 4,170 (68.48) |  |
| Body Mass Index, kg/m2, mean (SE) |  | 26.45 (4.90) | 26.48 (4.94) | 26.39 (4.81) | 0.564 |
| Body Mass Index, kg/m2, n (%) | Underweight (< 18.5) | 177 (0.97) | 122 (1.00) | 55 (0.90) | 0.847 |
|  | Normal (18.5 – 24.99) | 7,781 (42.47) | 5,185 (42.39) | 2,596 (42.63) |  |
|  | Overweight (25.0 – 29.99) | 6,971 (38.05) | 4,645 (37.97) | 2,326 (38.20) |  |
|  | Obese (≥ 30.0) | 3,393 (18.52) | 2,281 (18.65) | 1,112 (18.26) |  |
| BMI Waist Ratio, mean (SE) |  | 0.28 (0.03) | 0.28 (0.03) | 0.28 (0.03) | 0.277 |
| BMI Waist Ratio in Quartiles, mean (SE) | Quartile 1 | 0.25 (0.01) | 0.25 (0.01) | 0.25 (0.01) | 0.009 |
|  | Quartile 2 | 0.27 (0.01) | 0.27 (0.01) | 0.27 (0.01) | 0.818 |
|  | Quartile 3 | 0.29 (0.01) | 0.29 (0 .01) | 0.29 (0.01) | 0.251 |
|  | Quartile 4 | 0.32 (0.02) | 0.32 (0.02) | 0.32 (0.02) | 0.046 |
| Hip Circumference, mean (SE) |  | 104.85 (10.04) | 104.91 (10.13) | 104.73 (9.86) | 0.250 |
| Waist Circumference, mean (SE) |  | 92.40 (13.18) | 92.50 (13.29) | 92.20 (12.95) | 0.146 |
| Waist Circumference, n (%) | Normal (≤ 102 cm for male and ≤ 88 cm for female) | 10,319 (56.32) | 6,854 (56.03) | 3,465 (56.91) | 0.260 |
|  | Substantially increased risk of metabolic complications (> 102 cm for male and > 88 cm for female) | 8,003 (43.68) | 5,379 (43.97) | 2,624 (43.09) |  |
| Waist Hip Ratio, mean (SE) |  | 0.91 (0.07) | 0.91 (0.07) | 0.91 (0.07) | 0.882 |
| Waist Hip Ratio, n (%) | Normal (< 0.9 for male and < 0.85 for female) | 4,556 (24.87) | 3,056 (24.98) | 1,500 (24.63) | 0.609 |
|  | Abdominal obesity (≥ 0.9 for male and ≥ 0.85 for female) | 13,766 (75.13) | 9,177 (75.02) | 4,589 (75.37) |  |
| Body Fat Percentage, mean (SE) |  | 31.89 (8.62) | 31.93 (8.59) | 31.82 (8.68) | 0.411 |
| Body Fat Percentage, n (%) | Normal (< 25.0 for male and < 35.0 for female) | 9,386 (51.23) | 6,258 (51.16) | 3,128 (51.37) | 0.784 |
|  | Obese (≥ 25.0 for male and ≥ 35.0 for female) | 8,936 (48.77) | 5,975 (48.84) | 2,961 (48.63) |  |
| Diastolic Blood Pressure, mean (SE) |  | 72.95 (9.35) | 72.93 (9.35) | 72.97 (9.34) | 0.787 |
| Diastolic Blood Pressure, mmHg, n (%) | < 80 | 14,002 (76.42) | 9,373 (76.62) | 4,629 (76.02) | 0.533 |
|  | 80 – 89 | 3,467 (18.92) | 2,287 (18.70) | 1,180 (19.38) |  |
|  | ≥ 90 | 853 (4.66) | 573 (4.68) | 280 (4.60) |  |
| Systolic Blood Pressure, mean (SE) |  | 119.81 (13.73) | 119.75 (13.73) | 119.92 (13.71) | 0.446 |
| Systolic Blood Pressure, mmHg, n (%) | < 120 | 9,561 (52.18) | 6,398 (52.30) | 3,163 (51.95) | 0.245 |
|  | 120 – 129 | 4,561 (24.89) | 3,024 (24.72) | 1,537 (25.24) |  |
|  | 130 – 139 | 2,717 (14.83) | 1,846 (15.09) | 871 (14.30) |  |
|  | ≥ 140 | 1,483 (8.09) | 965 (7.89) | 518 (8.51) |  |
| Marital Status, n (%) | Married and/or living with a partner | 14,458 (78.91) | 9,659 (78.96) | 4,799 (78.81) | 0.226 |
|  | Single, never married | 1,180 (6.44) | 763 (6.24) | 417 (6.85) |  |
|  | Other (divorced, widowed, separated) | 2,684 (14.65) | 1,811 (14.80) | 873 (14.34) |  |
| Residence, n (%) | Urban | 15,272 (83.35) | 10,180 (83.22) | 5,092 (83.63) | 0.484 |
|  | Rural | 3,050 (16.65) | 2,053 (16.78) | 997 (16.37) |  |
| Total Household Income, n (%) | < $49,999 | 2,855 (15.58) | 1,904 (15.56) | 951 (15.62) | 0.416 |
|  | $50,000 - $99,999 | 5,889 (32.14) | 3,902 (31.90) | 1,987 (32.63) |  |
|  | $100,000 - $199,999 | 7,149 (39.02) | 4,823 (39.43) | 2,326 (38.20) |  |
|  | ≥ $200,000 | 2,429 (13.26) | 1,604 (13.11) | 825 (13.55) |  |
| Highest Education Level Completed, n (%) | High school or below (none, elementary school, high school, trade, technical or vocational school, apprenticeship training or technical CEGEP) | 6,161 (33.63) | 4,073 (33.30) | 2,088 (34.29) | 0.310 |
|  | Diploma but below bachelor’s degree (diploma from a community college, pre-university CEGEP or non-university certificate, university certificate below bachelor's level) | 4,928 (26.90 | 3,288 (26.88) | 1,640 (26.93) |  |
|  | Bachelor’s degree or above (bachelor's degree, graduate degree (MSc, MBA, MD, PhD, etc.)) | 7,233 (39.48) | 4,872 (39.83) | 2,361 (38.77) |  |
| Ethnicity, n (%) | Aboriginal | 68 (0.37) | 49 (0.40) | 19 (0.31) | 0.316 |
|  | Asian (South Asian, East Asian, Southeast Asian, Filipino, West Asian, Arab) | 827 (4.51) | 545 (4.46) | 282 (4.63) |  |
|  | White | 16,895 (92.21) | 11,274 (92.16) | 5,621 (92.31) |  |
|  | Latin American Hispanic | 162 (0.88) | 121 (0.99) | 41 (0.67) |  |
|  | Black | 97 (0.53) | 63 (0.52) | 34 (0.56) |  |
|  | Other (Jewish and others) | 273 (1.49) | 181 (1.48) | 92 (1.51) |  |
| Diabetes, n (%) |  | 735 (4.01) | 502 (4.10) | 233 (3.83) | 0.368 |
| Cardiovascular Disease, n (%) |  | 377 (2.06) | 257 (2.10) | 120 (1.97) | 0.559 |
| Depression, n (%) |  | 2,013 (10.99) | 1,366 (11.17) | 647 (10.63) | 0.270 |
| Family History of Hypertension, n (%) |  | 10,946 (59.74) | 7,266 (59.40) | 3,680 (60.44) | 0.176 |
| Smoking Status, n (%) | Never | 10,116 (55.21) | 6,739 (55.09) | 3,377 (55.46) | 0.763 |
|  | Former | 6,763 (36.91) | 4,537 (37.09) | 2,226 (36.56) |  |
|  | Current | 1,443 (7.88) | 957 (7.82) | 486 (7.98) |  |
| Ever Smoked, n (%) |  | 8,206 (44.79) | 5,494 (44.91) | 2,712 (44.54) | 0.633 |
| Alcohol Consumption, n (%) | Never | 1,293 (7.06) | 869 (7.10) | 424 (6.96) | 0.855 |
|  | ≤ 1 time a week | 9,644 (52.64) | 6,415 (52.44) | 3,229 (53.03) |  |
|  | 2 to 3 times a week | 3,807 (20.78) | 2,535 (20.72) | 1,272 (20.89) |  |
|  | 4 to 5 times a week | 1,993 (10.88) | 1,340 (10.95) | 653 (10.72) |  |
|  | ≥ 6 times a week | 1,585 (8.65) | 1,074 (8.78) | 511 (8.39) |  |
| Working Status, n (%) | Full time | 10,281 (56.11) | 6,836 (55.88) | 3,445 (56.58) | 0.065 |
|  | Part time | 3,719 (20.30) | 2,543 (20.79) | 1,176 (19.31) |  |
|  | Other (looking after home, disable/sick, student, unpaid/voluntary) | 3,974 (21.69) | 2,614 (21.37) | 1,360 (22.34) |  |
|  | Unemployed | 348 (1.90) | 240 (1.96) | 108 (1.77) |  |
| Total Sleep Time, n (%) | ≤ 5 hours (short sleep duration) | 1,191 (6.50) | 804 (6.57) | 387 (6.36) | 0.257 |
|  | 6 hours | 3,739 (20.41) | 2,441 (19.95) | 1,298 (21.32) |  |
|  | 7 hours (reference) | 7,042 (38.43) | 4,747 (38.80) | 2,295 (37.69) |  |
|  | 8 hours | 5,111 (27.90) | 3,414 (27.91) | 1,697 (27.87) |  |
|  | ≥ 9 hours (long sleep duration) | 1,239 (6.76) | 827 (6.76) | 412 (6.77) |  |
| Total Physical Activity Time, mean (SE) |  | 3158.53 (2869.02) | 3157.97 (2853.36) | 3159.66 (2900.45) | 0.970 |
| Total Physical Activity Time, n (%) | Light (< 450 MET minutes/week) | 1,668 (9.10) | 1,096 (8.96) | 572 (9.39) | 0.530 |
|  | Moderate (450 – 900 MET minutes/week) | 2,067 (11.28) | 1,394 (11.40) | 673 (11.05) |  |
|  | Vigorous (> 900 MET minutes/week) | 14,587 (79.61) | 9,743 (79.65) | 4,844 (79.55) |  |
| Total Sitting Time, mean (SE) |  | 2487.77 (1174.02) | 2495.39 (1176.80) | 2472.48 (1168.35) | 0.214 |
| Physical Activity, n (%) | Low (first quartile of physical activity time and fourth quartile of sitting time) | 1,691 (9.23) | 1,157 (9.46) | 534 (8.77) | 0.280 |
|  | Moderate (second and third quartile of physical activity time and sitting time) | 14,479 (79.03) | 9,653 (78.91) | 4,826 (79.26) |  |
|  | High (fourth quartile of physical activity and first quartile of sitting time) | 2,152 (11.75) | 1,423 (11.63) | 729 (11.97) |  |
| Vegetable and Fruit Consumption, n (%) | Low consumption (less than 5 servings of vegetable and fruit) | 15,273 (83.36) | 10,182 (83.23) | 5,091 (83.61) | 0.620 |
|  | Moderate consumption (less than 5 servings of vegetable but more than 5 servings of fruit OR more than 5 servings of vegetable but less than 5 servings of fruits | 2,529 (13.80) | 1,694 (13.85) | 835 (13.71) |  |
|  | High consumption (5 or more servings of vegetable and fruit) | 520 (2.84) | 357 (2.92) | 163 (2.68) |  |
| Job Schedule, n (%) | Regular daytime shift | 11,920 (65.06) | 7,985 (65.27) | 3,935 (64.62) | 0.385 |
|  | Other (evening shift, night shift, rotating shift, split shift, irregular shift, or on call) | 6,402 (34.94) | 4,248 (34.73) | 2,154 (35.38) |  |

**Table S3.** Baseline characteristics of study participants according to the missing status

| **Socio-demographic characteristics of groups** | | |  |  |
| --- | --- | --- | --- | --- |
| **Variable** |  | **Observations**  **(without missing values)** | **Observations**  **(imputed missing values)** | **P-value** |
| Age, years, mean (SE) |  | 50.99 (9.20) | 0 (0.00) |  |
| Sex, n (%) | Male | 5,763 (31.45) | 0 (0.00) |  |
|  | Female | 12,559 (68.55) | 0 (0.00) |  |
| Body Mass Index, kg/m2, mean (SE) |  | 26.40 (4.78) | 26.62 (5.27) | 0.009 |
| BMI Waist Ratio, mean (SE) |  | 0.28 (0.03) | 0.28 (0.03) | < 0.001 |
| Hip Circumference, mean (SE) |  | 104.80 (9.92) | 104.99 (10.41) | 0.257 |
| Waist Circumference, mean (SE) |  | 92.38 (13.14) | 92.44 (13.28) | 0.785 |
| Waist Hip Ratio, mean (SD) |  | 0.91 (0.07) | 0.91 (0.07) | 0.100 |
| Body Fat Percentage, mean (SE) |  | 31.90 (8.56) | 31.86 (8.79) | 0.795 |
| Diastolic Blood Pressure, mmHg, mean (SE) |  | 72.87 (9.36) | 73.22 (9.29) | 0.032 |
| Systolic Blood Pressure, mmHg, mean (SE) |  | 119.63 (13.71) | 120.41 (13.78) | 0.001 |
| Marital Status, n (%) | Married and/or living with a partner | 14,451 (78.90) | — | 0.392 |
|  | Single, never married | 1,180 (6.44) | 0 (0.00) |  |
|  | Other (divorced, widowed, separated) | 2,684 (14.65) | 0 (0.00) |  |
| Residence, n (%) | Urban | 15,272 (83.35) | 0 (0.00) |  |
|  | Rural | 3,050 (16.65) | 0 (0.00) |  |
| Total Household Income, n (%) | < $49,999 | 2,562 (15.14) | 293 (20.90) | < 0.001 |
|  | $50,000 - $99,999 | 5,427 (32.07) | 462 (32.95) |  |
|  | $100,000 - $199,999 | 6,649 (39.30) | 500 (35.66) |  |
|  | ≥ $200,000 | 2,282 (13.49) | 147 (10.49) |  |
| Highest Education Level Completed, n (%) | High school or below (none, elementary school, high school, trade, technical or vocational school, apprenticeship training or technical CEGEP) | 6,158 (33.63) | — | 0.769 |
|  | Diploma but below bachelor’s degree (diploma from a community college, pre-university CEGEP or non-university certificate, university certificate below bachelor’s level) | 4,924 (26.89) | — |  |
|  | Bachelor’s degree or above (bachelor's degree, graduate degree (MSc, MBA, MD, PhD, etc.)) | 7,229 (39.48) | — |  |
| Ethnicity, n (%) | Aboriginal | 68 (0.37) | 0 (0.00) | 0.978 |
|  | Asian (South Asian, East Asian, Southeast Asian, Filipino, West Asian, Arab) | 826 (4.51) | — |  |
|  | White | 16,873 (92.21) | 22 (95.65) |  |
|  | Latin American Hispanic | 162 (0.89) | 0 (0.00) |  |
|  | Black | 97 (0.53) | 0 (0.00) |  |
|  | Other (Jewish and others) | 273 (1.49) | 0 (0.00) |  |
| Diabetes, n (%) |  | 735 (4.01) | 0 (0.00) | 0.563 |
| Cardiovascular Disease, n (%) |  | 377 (2.06) | 0 (0.00) |  |
| Depression, n (%) |  | 2,009 (10.97) | — | 0.073 |
| Family History of Hypertension, n (%) |  | 10,946 (59.74) | 0 (0.00) |  |
| Smoking Status, n (%) | Never | 10,084 (55.17) | 32 (71.11) | 0.028 |
|  | Former | 6,755 (36.96) | — |  |
|  | Current | 1,438 (7.87) | — |  |
| Ever Smoked, n (%) |  | 8,197 (44.84) | — | 0.003 |
| Alcohol Consumption, n (%) | Never | 1,210 (6.92) | 83 (9.81) | 0.002 |
|  | ≤ 1 time a week | 9,177 (52.51) | 467 (55.20) |  |
|  | 2 to 3 times a week | 3,653 (20.90) | 154 (18.20) |  |
|  | 4 to 5 times a week | 1,909 (10.92) | 84 (9.93) |  |
|  | ≥ 6 times a week | 1,527 (8.74) | 58 (6.86) |  |
| Working Status, n (%) | Full time | 10,281 (56.11) | 0 (0.00) |  |
|  | Part time | 3,719 (20.30) | 0 (0.00) |  |
|  | Other (looking after home, disable/sick, student, unpaid/voluntary)6697 | 3,974 (21.69) | 0 (0.00) |  |
|  | Unemployed | 348 (1.90) | 0 (0.00) |  |
| Total Sleep Time, n (%) | ≤ 5 hours (short sleep duration) | 1,179 (6.52) | 12 (5.02) | 0.533 |
|  | 6 hours | 3,685 (20.38) | 54 (22.59) |  |
|  | 7 hours (reference) | 6,955 (38.46) | 87 (36.40) |  |
|  | 8 hours | 5,046 (27.90) | 65 (27.20) |  |
|  | ≥ 9 hours (long sleep duration) | 1,218 (6.74) | 21 (8.79) |  |
| Total Physical Activity Time, mean (SE) |  | 3168.50 (2866.67) | 2817.41 (2930.63) | 0.006 |
| Total Sitting Time, mean (SE) |  | 2493.30 (1174.18) | 2422.02 (1170.47) | 0.028 |
| Physical Activity, n (%) | Low (first quartile of physical activity time and fourth quartile of sitting time) | 1,449 (8.79) | 242 (13.11) | < 0.001 |
|  | Moderate (second and third quartile of physical activity time and sitting time) | 13,050 (79.21) | 1,429 (77.41) |  |
|  | High (fourth quartile of physical activity and first quartile of sitting time) | 1,977 (12.00) | 175 (9.48) |  |
| Vegetable and Fruit Consumption, n (%) | Low consumption (less than 5 servings of vegetable and fruit) | 15,031 (83.25) | 242 (90.98) | 0.004 |
|  | Moderate consumption (less than 5 servings of vegetable but more than 5 servings of fruit OR more than 5 servings of vegetable but less than 5 servings of fruits | 2,509 (13.90) | 20 (7.52) |  |
|  | High consumption (5 or more servings of vegetable and fruit) | 516 (2.86) | — |  |
| Job Schedule, n (%) | Regular daytime shift | 10,918 (77.88) | 1,002 (23.29) | < 0.001 |
|  | Other (evening shift, night shift, rotating shift, split shift, irregular shift, or on call) | 3,101 (22.12) | 3,301 (76.71) |  |

“—" indicates cell frequency < 10.

**Table S4.** Test of Cox proportional-hazards assumption

| **Variable** | **rho** | $\boldsymbol{\chi}^{\boldsymbol{2}}$ | **Degrees of freedom (df)** | **P-value** |
| --- | --- | --- | --- | --- |
| Sex | -0.06572 | 1.61 | 1 | 0.2049 |
| Total Physical Activity Time | 0.04143 | 0.54 | 1 | 0.4631 |
| Diabetes | -0.03620 | 0.54 | 1 | 0.4611 |
| Age | 0.04250 | 0.67 | 1 | 0.4121 |
| SBP | 0.00164 | 0.00 | 1 | 0.9731 |
| CVD | -0.05012 | 1.03 | 1 | 0.3109 |
| BMI | 0.05692 | 1.15 | 1 | 0.2826 |
| Age by BMI | -0.06566 | 1.59 | 1 | 0.2080 |
| Age by SBP | -0.01090 | 0.05 | 1 | 0.8167 |
| Age by Total Physical Activity Time | -0.04543 | 0.65 | 1 | 0.4208 |
| Age by Sex | 0.04340 | 0.74 | 1 | 0.3906 |
| Sex by SBP | 0.03560 | 0.47 | 1 | 0.4952 |
| Sex by CVD | 0.00310 | 0.00 | 1 | 0.9501 |
| Global Test |  | 9.66 | 13 | 0.7216 |

**APPENDIX 1.**

**Alberta’s Tomorrow Project.**

Alberta’s Tomorrow Project (ATP) is a part of a pan-Canadian initiative to investigate the causes and prevention of cancer and chronic diseases. Launched in 2000, ATP is Alberta’s largest longitudinal population health cohort from the general population. It contains baseline and longitudinal information on socio-demographic characteristics, personal and family history of the disease, medication use, lifestyle and health behavior, environmental exposures, and physical measures. ATP joined the Canadian Partnership for Tomorrow Project (CPTP) in 2008^1^. ATP had three baseline questionnaires: Canadian Diet History Questionnaire-I (CDHQ-I), Health and Lifestyle Questionnaire (HLQ), and the Past-Year Total Physical Activity Questionnaire (PYTPAQ), and two follow-up questionnaires: Survey 2004 and Survey 2008, during the period 2001-2008. When ATP merged with CPTP, participants were asked to complete two versions of questionnaires: The Updated Health and Lifestyle Questionnaire (UHLQ), along with the Physical Activity and Nutrition Survey (PANS) or the CORE questionnaire^2^. As both questionnaires contained very similar information, participants completed either UHLQ/PANS or CORE. UHLQ/PANS or CORE questionnaires were more elaborate and captured more information about the participants than the other questionnaires.

The recruitment of participants in ATP was done in two phases^3^. In Phase I (2000-08), participants were recruited using a two-stage telephone-based random digit dialing method^4^. Eight waves of telephone-based random digit dialing (RDD) using Alberta’s regional health authority boundaries as the sampling frame was used to recruit participants^4^. Participants were identified using a 2-stage method. In the first stage, a household was identified, and in the second stage, one or two eligible adults within the identified household were selected for participation^4^. Participants selected a second time from the same household were excluded to avoid repetition^3^. In Phase I, 29,878 participants were recruited with a response rate of 49%^3^.

In Phase II (2009-15), when ATP joined with the Canadian Partnership for Tomorrow Project (CPTP)–an alliance of five cohorts across Canada (British Columbia, Alberta, Ontario, Quebec, and Atlantic Canada), ATP-CPTP recruitment began using a volunteer sampling method^3^. Existing ATP participants (Phase I participants) were invited to join CPTP and requested to visit study centers for physical measurements and blood and urine contributions^3^. Fifteen thousand one hundred sixty-two participants from Phase I (approximately 50%) agreed to join CPTP, of which about 60% visited Study Centres^3^. Due to ATP’s pledge to enroll roughly 40,000 participants to CPTP from Alberta, more participants were recruited. Nevertheless, the process for selecting potential participants in CPTP varies between jurisdictions. It includes a random selection from population-based data, purchase of mailing lists for specific geographic areas, RDD, and word of mouth^5^. Telephone-based RDD was initially used to recruit new ATP-CPTP participants in 2009 but was soon replaced by volunteer sampling due to the low response rate and increasing cost^3^. To promote volunteer recruitment, further communication and advocating strategies were employed, such as marketing, advertising, media coverage, information booths at community events, corporate presentations, Ambassador Program, and articles^3^. In Phase II, 22,932 participants were recruited through volunteer sampling.

An invitation package was sent to the eligible participants (in both phases) that includes a cover letter, a study information booklet, an explicit consent to participate in the ATP and allow data linkage, and a self-administered ATP questionnaire^3^. Those who completed the ATP questionnaire and agreed to data linkage were considered as ATP participants. By March 2015, 52,810 Alberta residents had signed up for the ATP and decided to have their data linked to healthcare databases, with 38,094 of them agreeing to participate in the CPTP as well^3^. Of the total 52,810 ATP participants, 29,878 completed HLQ, 25,955 completed CDHQ, 25,889 completed PYTPAQ, 8,540 completed Survey 2004, 20,107 completed Survey 2008, 12,395 completed UHLQ, 12,402 completed PNAS and 25,677 completed the CORE questionnaire^3^.

**REFERENCES**

1. Summary Data Tables | Alberta’s Tomorrow Project. Accessed December 15, 2020. http://myatp.ca/for-researchers/summary-data-tables

2. Survey Questions Asked - Alberta’s Tomorrow Project. Accessed January 4, 2021. https://myatpresearch.ca/survey-questions/

3. Ye M, Robson PJ, Eurich DT, Vena JE, Xu JY, Johnson JA. Cohort profile: Alberta’s Tomorrow Project. *Int J Epidemiol*. 2017;46(4):1097-1098l. doi:10.1093/ije/dyw256

4. Robson PJ, Solbak NM, Haig TR, et al. Design, methods and demographics from phase I of Alberta’s Tomorrow Project cohort: a prospective cohort profile. *C Open*. 2016;4(3):E515-E527. doi:10.9778/cmajo.20160005

5. Borugian MJ, Robson P, Fortier I, et al. The Canadian Partnership for Tomorrow Project: Building a pan-Canadian research platform for disease prevention. *Cmaj*. 2010;182(11):1197-1201. doi:10.1503/cmaj.091540

**APPENDIX 2.**

**Description of the variables.**

The study participants’ age was categorized into four groups: 35 to less than 45, 45 to less than 55, 55 to less than 65, and greater than or equal to 65 years. Body mass index (BMI) was classified into four groups: underweight (< 18.5 kg/m2), normal (18.5 – 24.99 kg/m2), overweight (25.0 – 29.99 kg/m2), and obese (≥ 30.0 kg/m2). Waist circumference was classified as normal (≤ 102 cm for male and ≤ 88 cm for female) and substantially increased risk of metabolic complications (> 102 cm for male and > 88 cm for female) groups. The waist-hip ratio was categorized as normal (< 0.9 for male and < 0.85 for female) and abdominal obesity (≥ 0.9 for male and ≥ 0.85 for female). BMI waist ratio was categorized into four quartiles. Body fat percentage (BFP) was categorized as normal (< 25.0 for male and < 35.0 for female) and obese (≥ 25.0 for male and ≥ 35.0 for female). Diastolic blood pressure (DBP) was categorized into three groups: < 80 mm Hg, 80 – 89 mm Hg, and ≥ 90 mm Hg. Systolic blood pressure (SBP) was categorized into four groups: <120 mm Hg, 120 – 129 mm Hg, 130 – 139 mm Hg, and ≥ 140 mm Hg. Marital status was categorized into three groups: married and/or living with a partner, single who never married, and others (divorced, widowed, separated). Total household income was categorized into four groups: < $49,999, $50,000 - $99,999, $100,000 - $199,999, and ≥ $200,000. The highest education level completed was categorized into three groups: high school or below (none, elementary school, high school, trade, technical or vocational school, apprenticeship training or technical CEGEP), diploma but below bachelor’s degree (diploma from a community college, pre-university CEGEP or non-university certificate, university certificate below bachelor’s level), and bachelor’s degree or above (bachelor’s degree, graduate degree (MSc, MBA, MD, PhD, etc.). Ethnicity was categorized into six groups: Aboriginal, Asian (South Asian, East Asian, Southeast Asian, Filipino, West Asian, Arab), White, Latin American Hispanic, Black, and other (Jewish and others). Diabetes was categorized as “yes” or “no” based on the response to the question “Has a doctor ever told you that you had diabetes?”. Cardiovascular disease was categorized as “yes” if any stroke, myocardial infarction, angina, arrhythmia, coronary heart disease, coronary artery disease, heart disease, and heart failure was present and as ‘no” if absent. Depression was categorized as “yes” or “no” based on the response to the question “Has a doctor ever told you that you had depression?”. Family history of hypertension was categorized as “yes” if any first-degree relative is diagnosed with hypertension, otherwise “no”. Smoking status was categorized as: never, former, and current. Ever smoked was categorized as “yes” or “no” based on the response of the question “Have you smoked at least 100 cigarettes in your life?”. Alcohol consumption was categorized into five groups: never, ≤ 1 time a week, 2 to 3 times a week, 4 to 5 times a week, and ≥ 6 times a week. Working status was categorized into four groups: full-time, part-time, other (looking after a home, disable/sick, student, unpaid/voluntary), and unemployed. Total sleep time was categorized into four groups: ≤ 5 hours (short sleep duration), 6 to 7 hours, 8 hours, and ≥ 9 hours (long sleep duration). Total physical activity time was categorized as: light (< 450 MET minutes/week), moderate (450 – 900 MET minutes/week), and vigorous (> 900 MET minutes/week). Total sitting time was derived as the sum of the sitting times on weekdays and weekends. Physical activity was categorized as: low (first quartile of physical activity time and fourth quartile of sitting time), moderate (second and third quartile of physical activity time and sitting time), and high (fourth quartile of physical activity and first quartile of sitting time). Vegetable and fruit consumption was categorized as low (less than 5 servings of vegetable and fruit), moderate (less than 5 servings of vegetable but more than 5 servings of fruit OR more than 5 servings of vegetable but less than 5 servings of fruits), and high (5 or more servings of vegetable and fruit). Job schedule was categorized as regular daytime shift and other (evening shift, night shift, rotating shift, split shift, irregular shift, or on-call).
